# Bayesian-Optimized Collapse-Mode CMUT with Trenched Membrane for High Output Pressure

**DOI:** 10.3390/mi17080905

**Published:** 2026-07-29

**Authors:** Yuanyu Yu, Xin Liu, Jiujiang Wang, Shuang Zhang, Sio Hang Pun

**Affiliations:** 1 School of Artificial Intelligence, Neijiang Normal University, Neijiang 641100, China; cdyu@163.com (Y.Y.); zhang.s@njtc.edu.cn (S.Z.); 2 School of Mathematics and Computer Science, Northwest Minzu University, Lanzhou 730030, China; 3 Institute of Microelectronics, University of Macau, Macau 999078, China; lodgepun@um.edu.mo

**Keywords:** capacitive micromachined ultrasonic transducer (CMUT), collapse-mode CMUT, trenched membrane, Bayesian optimization, output acoustic pressure

## Abstract

Capacitive micromachined ultrasonic transducers (CMUTs) have been extensively investigated for applications in medical imaging and industrial non-destructive testing. However, their relatively low acoustic pressure output remains a major limitation to broader adoption. This paper proposes a CMUT structure that combines collapse-mode operation with a trenched membrane to enhance output performance. An analytical model based on von Kármán large-deflection plate theory is developed to estimate the optimal radial position of the trench, thereby defining the search space for subsequent Bayesian optimization. Single-parameter sequential Bayesian optimizations are first performed to identify the individual effects and optimal ranges of the trench’s radial position, depth, and width. Subsequently, a three-parameter global Bayesian optimization framework is employed for global parameter refinement. The three-parameter joint optimization reveals strong synergistic interactions among the design variables, achieving a higher output pressure of 72.34 kPa compared to 70.02 kPa from sequential approaches. Under identical operating conditions, the optimized trenched membrane CMUT exhibits a 100.15% increase in output acoustic pressure and a 28.77% improvement in the pressure-bandwidth product compared to a uniform membrane. Statistical analysis across multiple independent runs yielded a coefficient of variation (CV) of only 0.052% for the output pressure in the three-parameter global optimization results. This confirms that the proposed framework robustly optimizes CMUT designs for high output pressure, offering a promising technical approach to enhancing device performance.

## 1. Introduction

CMUTs are devices that utilize electrostatic actuation to convert electrical energy into acoustic energy and vice versa. They are typically fabricated using silicon-based micro-electro-mechanical systems (MEMSs) techniques, representing a significant shift in ultrasonic transducer technology from traditional piezoelectric materials to semiconductor integration. Compared to piezoelectric ultrasonic transducers like lead zirconate titanate (PZT), CMUTs offer advantages such as high bandwidth and the ability to be integrated with CMOS circuits on a single chip. CMUTs have been explored in nearly all ultrasound applications [1].

Despite significant advancements in CMUT technology, there remain some challenges in its applications. One limitation is the relatively low output acoustic pressure of CMUTs in transmission mode, which restricts their use in medical ultrasound imaging and can compromise image quality. For example, Perez et al. conducted a cross-sectional study on handheld ultrasound devices for point-of-care ultrasound (POCUS), where 35 physicians compared ultrasound imaging of the right upper quadrant of the abdomen, the apical four-chamber view of the heart, as well as superficial views of the neck and lungs [2]. In their comparative study, it was found that the handheld ultrasound device Butterfly iQ+, which uses CMUT technology, scored lowest across imaging quality metrics, including detail resolution, contrast resolution, penetration, and clutter. It is mainly caused by the relative low output acoustic pressure of CMUTs. Therefore, increasing the output acoustic pressure of CMUTs is a research direction to enhance their performance.

The output acoustic pressure of CMUTs is positively correlated with the membrane vibration amplitude. Theoretically, increasing the cavity height of the CMUT can enhance the output acoustic pressure; however, this approach may result in a decrease in receiving sensitivity. Currently, the methods to improve output acoustic pressure mainly include altering the operating mode, modifying the device structure, or combining both approaches. In both transmission and reception modes, CMUTs require an external DC bias voltage to operate at their working point. One way to enhance the output acoustic pressure of CMUTs is to change the applied DC bias voltage, allowing the device to enter unconventional operating modes. Research has shown that the collapse-snapback mode [3], collapse-mode [4,5,6], and deep-collapse mode [7] can all effectively enhance the output acoustic pressure of CMUTs. Among these operation modes, only the collapse-mode can improve both the transmission and reception performance of the CMUT while also increasing the bandwidth [8].

Collapse-mode refers to the operational state of a CMUT in which the applied DC bias voltage remains consistently above the pull-in voltage. In this mode, the central part of the membrane is in contact with the insulating layer of the substrate, while the annular region of the membrane that has not collapsed is responsible for transmitting and receiving ultrasound waves. In the white paper published by Health.E Lighthouse in 2021, several companies and institutions in Europe participated in benchmark testing of MEMS ultrasound transducers, including conventional CMUTs, collapse-mode CMUTs, and piezoelectric micromachined ultrasonic transducers (PMUTs). The results indicated that the collapse-mode CMUTs exhibited the highest peak-to-peak pressure and demonstrated the best overall acoustic performance, along with comparable round-trip sensitivity [9]. Consequently, collapse-mode represents a promising operational mode for enhancing the output acoustic pressure of CMUTs.

The output acoustic pressure of a CMUT is generated by the vibration of the membrane. Therefore, increasing the displacement during membrane vibration can lead to a higher output pressure. Since the edges of the membrane are fixed to the support, the amplitude of vibration is maximized at the center of the membrane, while the amplitude near the edges approaches zero. As a result, the average displacement of the membrane’s vibration is constrained by this structure. The output acoustic pressure can also be improved by modifying the structure of the CMUT to enable piston-like vibration of the membrane. One approach involves designing a non-uniform membrane that alters the membrane structure to achieve this piston-like motion. This design specifically includes features such as a center-mass and trench structures in the membrane. The center-mass method involves introducing a mass at the location of maximum amplitude of the membrane, which is at the center. This structure increases the local inertia of the membrane, facilitating the maintenance of coherent motion throughout the membrane. As a result, the piston-like mode becomes the dominant mode, leading to improvements in the output acoustic pressure [10,11]. Additionally, incorporating trench structures into the membrane can modify its stiffness, allowing the membrane to move more freely as a whole during applied forces in a piston-like manner. It enhances the volume displacement of the membrane and consequently increases the output acoustic pressure. Zhou et al. conducted a study using finite element analysis (FEA) to investigate the design of annular trenches on the membrane of conventional CMUTs. This approach can increase the membrane displacement, leading to more uniform deformation and ultimately enhancing the output acoustic pressure [12]. In their study, the output acoustic pressure of the CMUT with a single trench-structured membrane was improved by 14.4%. Jiang et al. investigated the reception performance of trench-structured membrane CMUTs, finding that a single trench structure improved the reception performance by 38.3% [13]. Their work indicates that trench-structured membranes can simultaneously enhance both the transmission and reception performance of CMUTs.

In addition to non-uniform membrane techniques, enhancements can be achieved by modifying the electrode structure or cavity design to increase the acoustic output pressure. Guldiken et al. proposed a dual-top-electrode structure for CMUTs to enhance the output acoustic pressure. The principle of this design is to utilize the lever bending effect to increase the effective vertical displacement of the membrane and the overall volume displacement, thereby improving the output acoustic pressure [14,15]. Li et al. introduced a ring-shaped electrode structure that employs the electrostatic spring softening effect to alter the overall stiffness of the membrane. This modification causes the membrane to vibrate with piston-like deformation when subjected to force, thereby enhancing the output acoustic pressure [16]. Li et al. proposed a T-shaped cavity structure for CMUTs [17], while Jia et al. introduced “2-stage-Optimal” and “3-stage” cavity structures for CMUTs [18]. These methods all incorporate stepped cavity designs, which increase the electric field strength at the edges of the membrane. Additionally, they utilize the electrostatic stiffness softening effect to adjust the membrane’s stiffness, allowing the entire membrane to undergo piston-like deformation, thereby enhancing the output acoustic pressure. Additionally, Yuan et al. introduced a design method that combines an annular electrode with a membrane groove to enhance the output acoustic pressure of CMUTs. The annular electrode utilizes the electrostatic stiffness softening effect to partially adjust the stiffness of the membrane, while the groove effectively releases stress at the edges of the membrane, promoting piston-like deformation and increasing the average displacement [19]. Kim et al. designed a circular membrane and electrode structure with a separated suspension. By employing an indirectly clamped method, the circular membrane experiences piston-like deformation under electrostatic forces, thereby enhancing the output acoustic pressure [20]. Li et al. proposed a dual-layer CMUT design method that stacks a top circular CMUT cell on top of a bottom annular CMUT cell. This configuration reduces stiffness and allows the deflections of both layers to combine, making it easier for the membrane to deform under external forces and thus improving the output acoustic pressure [21]. Our research group previously proposed a method that combines collapse-mode operation with a center-mass design, referred to as an embossed pattern, to enhance the output acoustic pressure of CMUTs. Specifically, an annular mass structure is added at the center of vibration of the collapsed membrane to form the embossed pattern. Both FEA simulations and device experiments have demonstrated that this method can further increase the output acoustic pressure of collapse-mode CMUTs [22,23].

Previous research has demonstrated that introducing trench structures on CMUT membranes can enhance output acoustic pressure. This study extends this concept to collapse-mode CMUTs by incorporating a trench into the vibrating membrane. To the best of the authors’ knowledge, the integration of collapse-mode operation with three-parameter trench-geometry optimization has not been reported previously. The primary contributions of this work are as follows: Firstly, the vibrating membrane of the collapse-mode CMUT is analyzed using the von Kármán large-deflection plate theory to analytically estimate the optimal radial position of the trench. Secondly, a three-parameter Bayesian optimization framework is implemented to globally optimize the radial position, depth, and width of the trench. Thirdly, the derived analytical criterion is utilized to constrain the search space for Bayesian optimization, thereby effectively coupling physical modeling with a data-driven method while enhancing computational efficiency. This paper is organized as follows. Section 2 describes the trenched membrane CMUT design, FEA models, and analysis methods. Section 3 presents the validation of the proposed approach. Section 4 reports the optimization process and results. Section 5 discusses the findings, and Section 6 concludes the paper.

## 2. Methodology

### 2.1. Conception of a Collapse-Mode CMUT with Trenched Membrane

**Figure 1:**
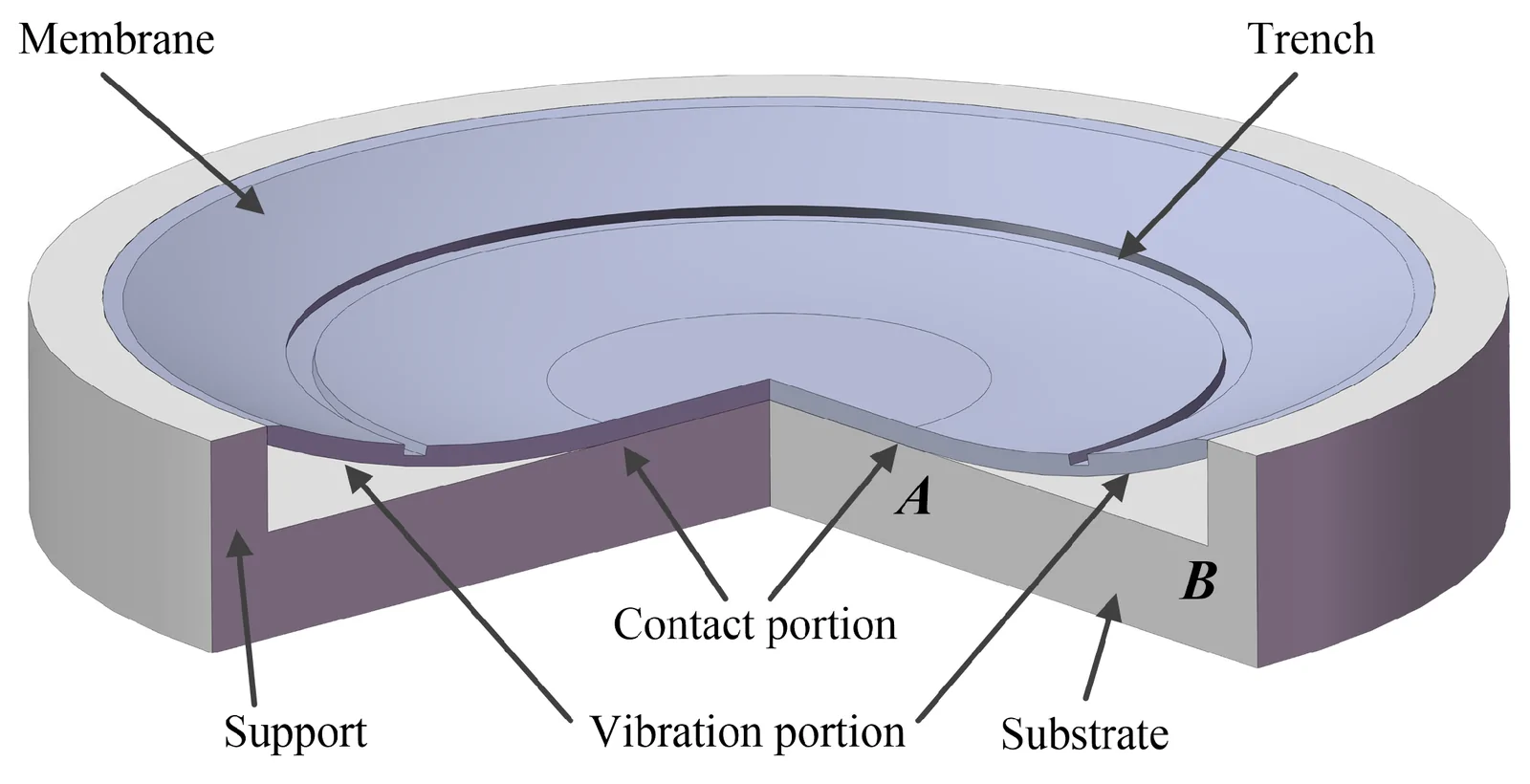
Three-dimensional outlook view of the collapse-mode CMUT with trenched membrane. *A* denotes the contact point of membrane on substrate, and *B* denotes the edge of membrane on the support.

CMUTs are electrostatic transducers that operate by applying a DC bias voltage to deflect the membrane, followed by the superposition of an AC voltage or pulse to drive membrane vibration, thereby generating acoustic pressure. In conventional CMUTs, the applied DC bias voltage is maintained below the pull-in voltage. Once the DC bias voltage exceeds the pull-in voltage, the membrane collapses and comes into contact with the substrate insulating layer. The uncollapsed annular region of the membrane surrounding the collapsed area then vibrates to generate acoustic pressure. A trench structure is introduced into the annular vibrating membrane to improve acoustic pressure, as shown in Figure 1. Whether for a circular vibrating membrane in conventional mode or an annular vibrating membrane in collapse mode, the output acoustic pressure of CMUTs is inherently related to the amplitude of the membrane’s vibration. As mentioned above, the design of the trench structure reduces the local stiffness of the vibrating membrane, thereby increasing the volume displacement of the membrane and enhancing the output acoustic pressure.

### 2.2. Analytical Analysis of Optimal Trench Position

To achieve the maximum increase in acoustic pressure, it is essential to investigate the optimal placement of the trench. In previous studies of conventional mode CMUTs, trenches are typically designed near the edge of the membrane [12,13,24]. However, since the vibrating membrane in collapse-mode CMUTs differs substantially from that in conventional-mode CMUTs, it is necessary to investigate the optimal trench position to enhance acoustic pressure, taking into account the characteristics of collapse-mode operation.

#### 2.2.1. Analytical Model for Optimal Trench Position

**Figure 2:**
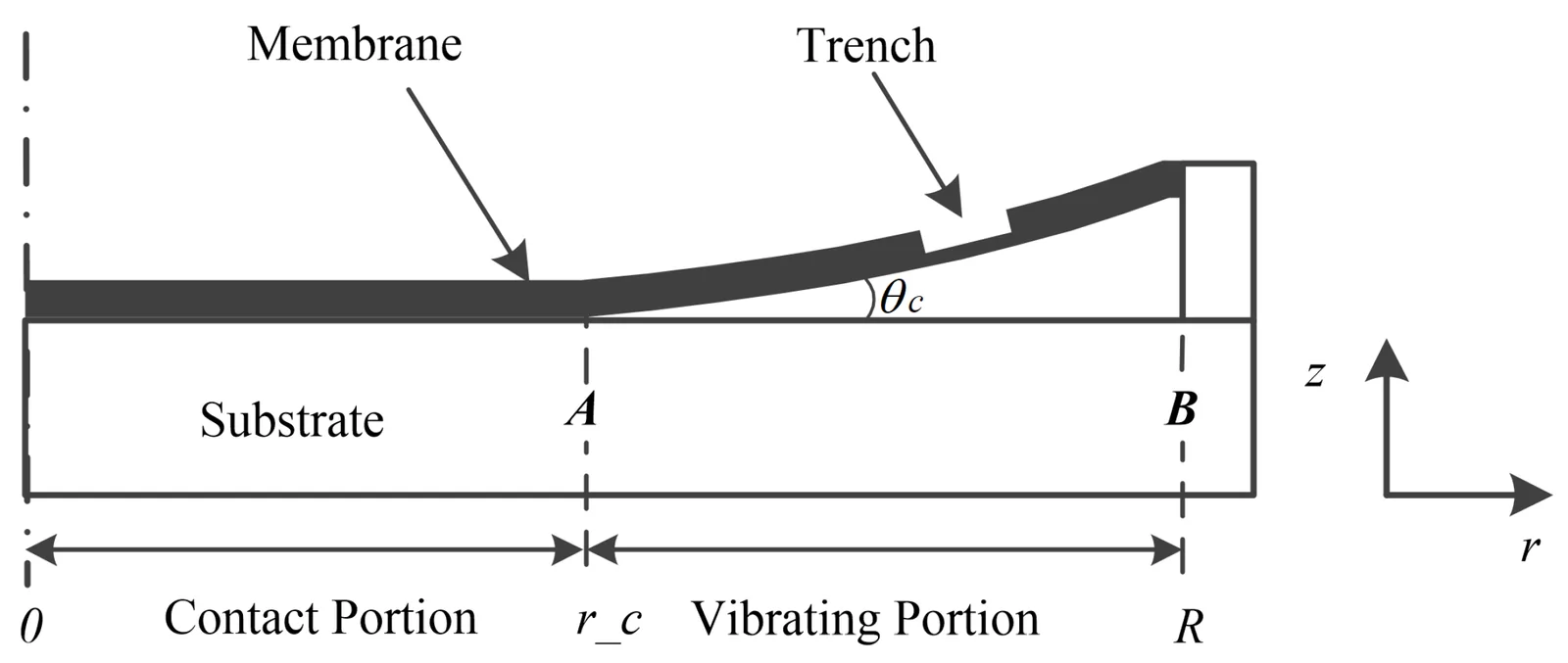
Two-dimensional axisymmetric cross-sectional view the collapse-mode CMUT with trenched membrane (The schematic is for illustrative purposes only). *R* is radius of membrane, r_c is the contact radius of membrane, *A* denotes the contact point of membrane on substrate, and *B* denotes the edge of membrane on the support.

Figure 2 shows the cross-section view of a CMUT with trenched membrane in collapse-mode. The membrane is divided into two sections; the inner contact portion is constrained by the strong electrostatic force at the insulating layer of the substrate, as indicated at point *A* in Figure 2. The outer vibrating portion remains uncollapsed, which is fixed to the support, as shown at point *B*. In the transmitting mode, only the uncollapsed membrane vibrates. Therefore, the analysis focuses on the vibrating portion of the membrane. The dynamic behavior of the membrane is analyzed using von Kármán large-deflection plate theory, which accounts for geometric nonlinearity in the static deflection. For the small-signal AC dynamic response, the system is linearized around the statically deformed state. The amplitude of the AC voltage is small compared to the DC bias, and the residual stress in the membrane is neglected. This analysis helps estimate the optimal radial position of the trench and narrows the search space for Bayesian optimization.

For the collapse-mode CMUT, the overall radius of the membrane is denoted as *R*. Under the influence of a DC bias voltage, the central region of the membrane is closely adhered to the substrate, forming a fixed contact area. The radius of the contact boundary is represented as r_c, which is uniquely determined by the DC bias voltage. The effective vibrating area of the membrane is limited to the annular non-contact region (r_c<r<R), and the central contact area cannot vibrate, thus not contributing to acoustic wave radiation. The output acoustic pressure of a collapse-mode CMUT depends on the total volume velocity of the entire non-contact area, defined as the spatial integral of the vibrational velocity over the effective radiation area. This can be mathematically expressed as (1).

(1) $$Q=\underset{S}{\;\int \int \;}v\;dS$$

where *Q* represents the total volume velocity, *v* is the normal particle vibration velocity of the membrane, and *S* is the effective radiation area of the membrane in the non-contact zone. The expression of *v* is as follows (2):

(2) v=jωw(r)

where w(r) is the normal dynamic deflection of the membrane in the annular region under the action of electrostatic forces. In Equation (1), the differential area element of the annular region is given by dS=2πrdr. Therefore, the two-dimensional area integral can be simplified to a one-dimensional radial integral: (3).

(3) $$Q=j\omega \int_{r\text{\_}c}^{R}w\left(r\right)\;\cdot \;2\pi r\;dr$$

In low-frequency small-signal radiation conditions, the output acoustic pressure of the CMUT is positively correlated with *Q*. Therefore, the essence of enhancing the output acoustic pressure is to maximize the integral of *Q*. The radially weighted integrand function of the volume velocity, I(r), is defined as (4).

(4) I(r)=2πrw(r)

This function incorporates both the local membrane vibration deflection w(r) and radius *r*. The peak position of I(r) represents the location within the region where the contribution to acoustic radiation is the strongest. Because a trench reduces the bending stiffness of the membrane through localized thinning, it selectively amplifies local vibration amplitude. Thus, placing the trench structure at the peak position of I(r) can maximize the effective radiation contribution and achieve optimal enhancement of the output acoustic pressure. This position corresponds to the theoretically optimal radial location for enhancing the acoustic pressure of the trenched collapse-mode CMUT. The trench modifies the stiffness distribution and the deflection profile w(r) of the membrane, which can be analyzed using the dynamic equations of von Kármán’s large deflection theory [25,26], as shown in (5) and (6).

(5) $$\rho h\frac{\partial^{2}\;w\left(r\right)}{\partial t^{2}}+D\nabla^{4}\;w=\frac{1}{r}\frac{\partial^{2}\;w\left(r\right)}{\partial r^{2}}\frac{\partial F}{\partial r}+\frac{1}{r}\frac{\partial w(r)}{\partial r}\frac{\partial^{2}\;F}{\partial r^{2}}+F_{e}$$

(6) $$\nabla^{4}\;F=-\frac{Eh}{r}\frac{\partial^{2}\;w\left(r\right)}{\partial r^{2}}\frac{\partial w(r)}{\partial r}$$

where ρ is the density of membrane material; *h* denotes the thickness of membrane; *D* represents the bending stiffness of membrane defined in (8); and *E* represents the Young’s modulus of the membrane material. $\nabla^{4}\;w\left(r\right)$ is the biharmonic operator described in (9). $F_{e}$ denotes the electrostatic force defined in (10). *F* is the axisymmetric Airy stress function, and its relationship with the radial membrane stress $N_{r}$ and tangential membrane stress $N_{\theta }$ is as (7).

(7) $$N_{r}=\frac{1}{r}\frac{\partial F}{\partial r},\;N_{\theta }=\frac{\partial^{2}\;F}{\partial r^{2}}$$

(8) $$D=\frac{h^{3}}{12\left(1-\nu^{2}\right)}$$

where ν is the Poisson’s ratio of the membrane material. Both of these properties are determined by the specific material used for the membrane.

(9) $$\nabla^{4}\;w\left(r\right)=\frac{d^{4}\;w}{dr^{4}}+\frac{2}{r}\frac{d^{3}\;w}{dr^{3}}-\frac{1}{r^{2}}\frac{d^{2}\;w}{dr^{2}}+\frac{1}{r^{3}}\frac{dw}{dr}$$

(10) $$F_{e}=\frac{\varepsilon_{0}\;V_{\mathrm{dc}}^{2}}{2{\left[d_{0}-w\left(r\right)\right]}^{2}}$$

In (10), $\varepsilon_{0}$ represents the vacuum permittivity, $V_{dc}$ is the DC bias voltage, and $d_{0}$ is the initial gap height between the plates when the membrane is undeformed. Under a DC bias voltage, the membrane collapses and contacts the bottom, forming a stable static operating point with a large static deflection of $w_{\mathrm{dc}}\left(r\right)$. At this point, removing the time term, Equation (5) reduces to the large static deflection equation in (11).

(11) $$D\nabla^{4}\;w_{\mathrm{dc}}\left(r\right)=\frac{1}{r}\frac{dw_{\mathrm{dc}}\left(r\right)}{dr}\frac{d^{2}\;F_{\mathrm{dc}}}{dr^{2}}+\frac{1}{r}\frac{d^{2}\;w_{\mathrm{dc}}\left(r\right)}{dr^{2}}\frac{dF_{\mathrm{dc}}}{dr}+F_{e,\mathrm{dc}}$$

Combining the compatibility Equation (6), the static axisymmetric Airy stress function $F_{dc}$ can be obtained, and then the static membrane force $N_{0}$, which includes all geometric nonlinear effects caused by large deformation in the collapse mode, can be obtained in (12).

(12) $$N_{0}=\frac{1}{r}\frac{dF_{\mathrm{dc}}}{dr}$$

In collapse-mode, the AC excitation voltage is smaller than the DC bias voltage. Therefore, the AC small-signal excitation can be regarded as a small dynamic perturbation on a static large deflection basis, and the perturbation method can be used for research. The total displacement *w* and the axisymmetric Airy stress function can be decomposed as in (13).

(13) $$\begin{array}{cc} w(r,t) & =w_{\mathrm{dc}}\left(r\right)+w_{\mathrm{ac}}\left(r,t\right),\;w_{\mathrm{ac}}\ll w_{\mathrm{dc}}\\ F(r,t) & =F_{\mathrm{dc}}\left(r\right)+F_{\mathrm{ac}}\left(r,t\right),\;F_{\mathrm{ac}}\ll F_{\mathrm{dc}} \end{array}$$

By substituting the decomposition into (5), the nonlinear terms are expanded. Only first-order terms of $w_{ac}$ and $F_{ac}$ are kept, whereas higher-order terms are omitted. The relationship between $F_{dc}$ and $w_{ac}$ is established via the linearization of the compatibility (6), resulting in a linearized dynamic equation involving only $w_{ac}$ as in (14).

(14) $$D\nabla^{4}\;w_{\mathrm{ac}}-\nabla \;\cdot \;\left(N_{0}\nabla w_{\mathrm{ac}}\right)+\rho h\frac{\partial^{2}\;w_{\mathrm{ac}}}{\partial t^{2}}=F_{\mathrm{ac}}$$

The stress concentration induced by contact is highly localized near the contact edge of $r_{c}$ and decays rapidly into the non-contact region. Within the interior annular region, the membrane stress $N_{0}$ can be treated as slowly varying. Since the objective of this study is to locate the peak radial position of the volume velocity integrand of I(r) rather than to calculate the absolute membrane amplitude, the Rayleigh-Ritz modal approximation indicates that uniform or slowly varying inertial forces and prestress primarily influence the vibration amplitude. In contrast, the locations of the peak of the spatial mode shape functions are constrained by geometric boundary conditions [27]. Consequently, for the estimation of the optimal radial position of the trench, the governing equations reduce to a homogeneous form dictated solely by geometric boundary conditions, thereby allowing the boundary-driven deformation to be described by the general solution of (15) without the need for a separate particular solution.

(15) $$D\nabla^{4}\;w\left(r\right)=0$$

Since D≠0, the deformation shape is governed by $\nabla^{4}\;w\left(r\right)=0$. This homogeneous equation determines the radial position of the displacement peak, while the absolute deflection amplitude is not predicted by this simplified model. w(r) is a quartic function of *r*, with its boundary conditions given by (16) and (17). At the outer boundary *B*, it is fixed-supported, while at the inner boundary *A*, it is adhered due to the electrostatic force. The contact angle at the edge is $\theta_{c}$. These four boundary conditions constrain the fourth-order differential governing equation of the annular CMUT diaphragm, enabling the analytical solution of radial deflection distribution w(r).

(16) $$\begin{array}{cc} \; & \mathrm{Outer}\;\mathrm{boundary}\;\mathrm{of}\;\mathrm{point}\;B:\left\{\begin{array}{c} w(R)=0\\ {\left.\frac{dw}{dr}\right|}_{r=R}=0 \end{array}\right. \end{array}$$

(17) $$\begin{array}{cc} \; & \mathrm{Inner}\;\mathrm{boundary}\;\mathrm{of}\;\mathrm{point}\;A:\left\{\begin{array}{c} w(r\text{\_}c)=0\\ {\left.\frac{dw}{dr}\right|}_{r=r\text{\_}c}=\theta_{c} \end{array}\right. \end{array}$$

The solution of (15) is dominated by the boundary conditions specified in (16) and (17), and it represents the standard general solution for an axisymmetric annular plate, as in (18). The four undetermined coefficients A,B,C,D are uniquely solved from the boundary conditions.

(18) $$w\left(r\right)=A+Br^{2}+C\ln r+Dr^{2}\ln r$$

(18) consists of four independent deformation components: constant term A represents rigid-body translation; quadratic term $Br^{2}$ corresponds to uniform spherical bending; logarithmic term Clnr characterizes local slope mutation induced by inner-edge electrostatic adhesion; the coupled term $Dr^{2}\ln r$ reconciles deformation transition between inner tilted boundary and outer clamped boundary. To simplify the calculations, a normalization process as in (19) is applied to eliminate the influence of radius.

(19) $$\eta =\frac{r}{R};\;\eta_{c}=\frac{r\text{\_}c}{R}$$

where η represents the dimensionless normalized radius, which ranges from $\eta_{c}$ to 1, where $\eta_{c}$ is the dimensionless collapse contact ratio determined by the DC bias voltage under the collapse-mode. The normalized deflection w(η) and the boundary conditions are given by (20) and (21), respectively.

(20) $$w\left(\eta \right)=\theta_{c}R\;\cdot \;f\left(\eta ,\eta_{c}\right)$$

(21) $$\left\{\begin{array}{c} f(\eta_{c})=0\\ f^{\prime }\left(\eta_{c}\right)=1\\ f(1)=0\\ f^{\prime }\left(1\right)=0 \end{array}\right.$$

w(r) is the actual deflection field expressed as a function of the physical radius *r*, while w(η) represents the same deflection distribution transformed through the normalized coordinate $\eta =\frac{r}{R}$. The two are related by this variable substitution. Additionally, $f(\eta ,\eta_{c})$ is the dimensionless curvature shape function. Therefore, (4) can be transformed into (22).

(22) $$I\left(\eta \right)=w\left(\eta \right)\;\cdot \;2\pi \left(\eta R\right)\;\cdot \;R=2\pi R^{3}\theta_{c}\;\cdot \;f\left(\eta ,\eta_{c}\right)\eta$$

The condition for I(η) to reach its maximum value is that its derivative equals zero, and the general solution of the dimensionless shape function is (23).

(23) $$f\left(\eta \right)=A_{0}+B_{0}\eta^{2}+C_{0}\ln\eta +D_{0}\eta^{2}\ln\eta$$

where $A_{0},B_{0},C_{0},D_{0}$ are undetermined constants. Substituting the normalized boundary conditions into the shape function and its first-order derivative yields a system of linear algebraic equations with respect to the undetermined coefficients $A_{0},B_{0},C_{0},$ and $D_{0}$, which can be expressed in matrix form as (24).

(24) $$\left[\begin{array}{cccc} 1 & \eta_{c}^{2} & \ln\eta_{c} & \eta_{c}^{2}\ln\eta_{c}\\ 0 & 2\eta_{c} & \frac{1}{\eta_{c}} & \eta_{c}\left(1+2\ln\eta_{c}\right)\\ 1 & 1 & 0 & 0\\ 0 & 2 & 1 & 1 \end{array}\right]\left[\begin{array}{c} A_{0}\\ B_{0}\\ C_{0}\\ D_{0} \end{array}\right]=\left[\begin{array}{c} 0\\ 1\\ 0\\ 0 \end{array}\right]$$

To solve the fourth-order linear system of equations, the coefficients $A_{0}$, $B_{0}$, $C_{0}$, and $D_{0}$ can be determined by applying the given boundary conditions. By substituting the coefficients into $f(\eta ,\eta_{c})$ and performing differentiation, the peak position $\eta^{*}$ can be determined. This position is the optimal location for placing the trench. When $\eta_{c}=0.3$, $\eta^{*}\approx 0.62$; when $\eta_{c}=0.4$, $\eta^{*}\approx 0.67$; when $\eta_{c}=0.5$, $\eta^{*}\approx 0.72$; and when $\eta_{c}=0.6$, $\eta^{*}\approx 0.77$. Based on this data, the peak position appears to be located near $(\eta_{c}+1)/2$, which is slightly outside the midpoint between the contact edge and the support. Therefore, the optimal radial coordinate $r_{1}^{*}$ can be approximately estimated using (25). This position represents the ideal location for placing the trench to enhance the output acoustic pressure.

(25) $$r_{1}^{*}\approx \frac{r\text{\_}c+R}{2}$$

From the analysis above, it is evident that the optimal position for placing a trench to enhance the output acoustic pressure of CMUT in the collapse-mode is different from that in the conventional mode of the CMUT, which is suggested to be placed near the rim.

#### 2.2.2. Applicability of the Analytical Model for Position Prediction

To evaluate the effectiveness of the proposed method in estimating the peak location of I(r), comparisons are performed using a finite element (FE) CMUT model with a uniform membrane, which is to be described in Section 2.3. In this study, the radius of the CMUT membrane is 25 μm. The prestressed analysis is employed, where a DC bias voltage exceeding the pull-in voltage is first applied to the collapse membrane, followed by the superimposition of an AC small-signal frequency sweep to analyze the vibration amplitude and output pressure. By varying the DC bias, the collapse radius can be adjusted, thereby modifying the width of the annular vibrating region.

**Figure 3:**
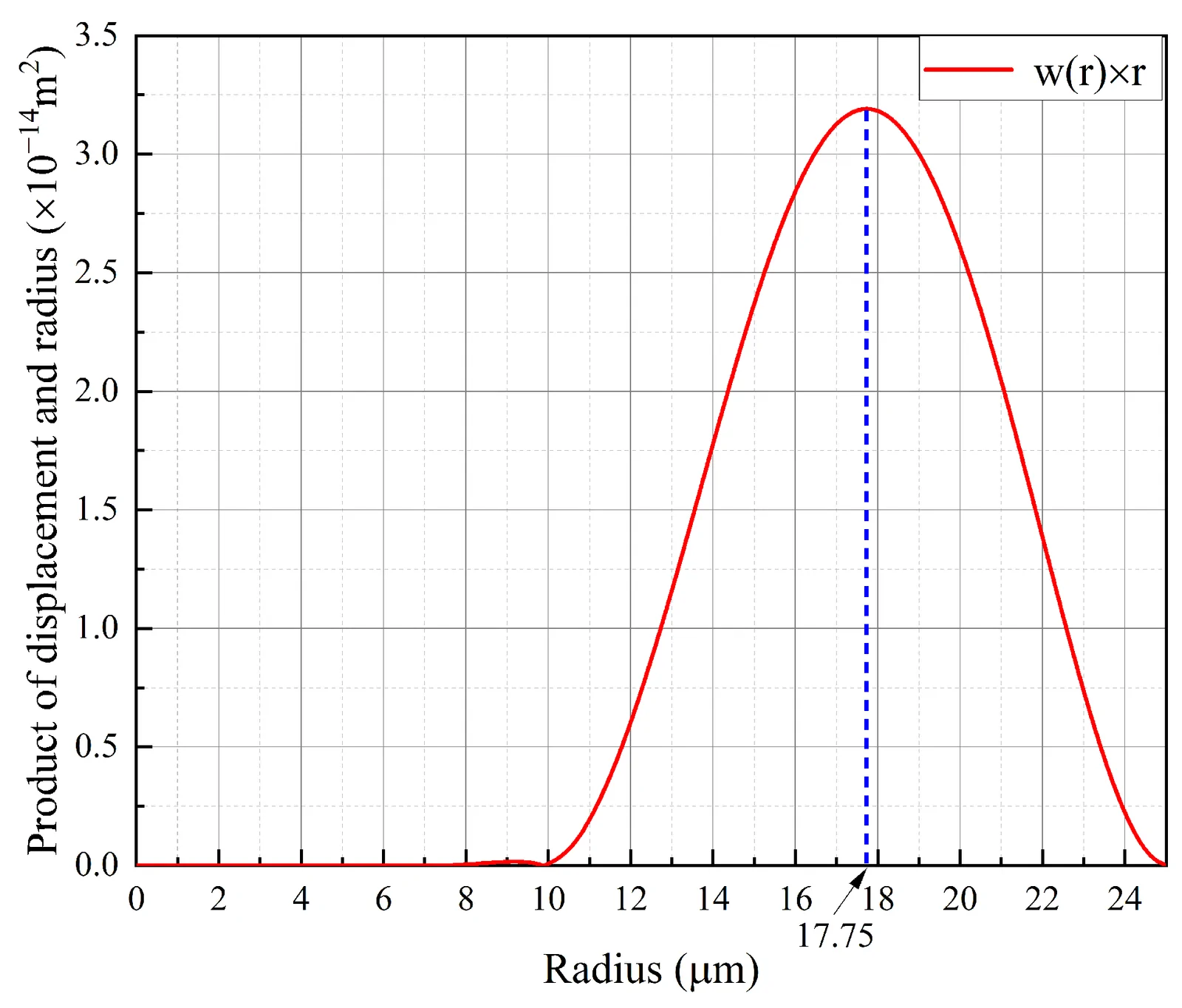
Radial profile of the weighted displacement w(r)·r for optimal trench placement. The blue dashed line indicates the peak position at *r* = 17.75 μm.

Figure 3 is the curve of w(r)·r in the volume velocity function I(r) at the center frequency, for the collapse radius of 10 μm and the vibrating membrane width of 15 μm, corresponding to $\eta_{c}=0.4$. According to the estimate from (25), the value of $r_{1}^{*}$ is 17.50μm, while the peak location from the FEA simulation is 17.75μm. The error in the analytical estimation is calculated to be −1.41%. Table 1 presents the analytical estimated optimum positions and the corresponding FEA simulation results for different collapse radii ($\eta_{c}=0.3,0.4,0.5,\mathrm{and}\;0.6$), with all errors remaining below 3%. It shows that the estimation positions of (25) is effective within this range and can be utilized for estimating the optimal trench radial position.

However, (25) provides a theoretical analysis result that does not take into account the geometric parameters of the trench. Additionally, the introduction of a trench structure may cause a slight change in the contact radius r_c. Therefore, it is necessary to use FEA to further analyze the optimal position for the trench, as well as to investigate the best dimensions for the trench at that position.

### 2.3. FEA Analysis

**Figure 4:**
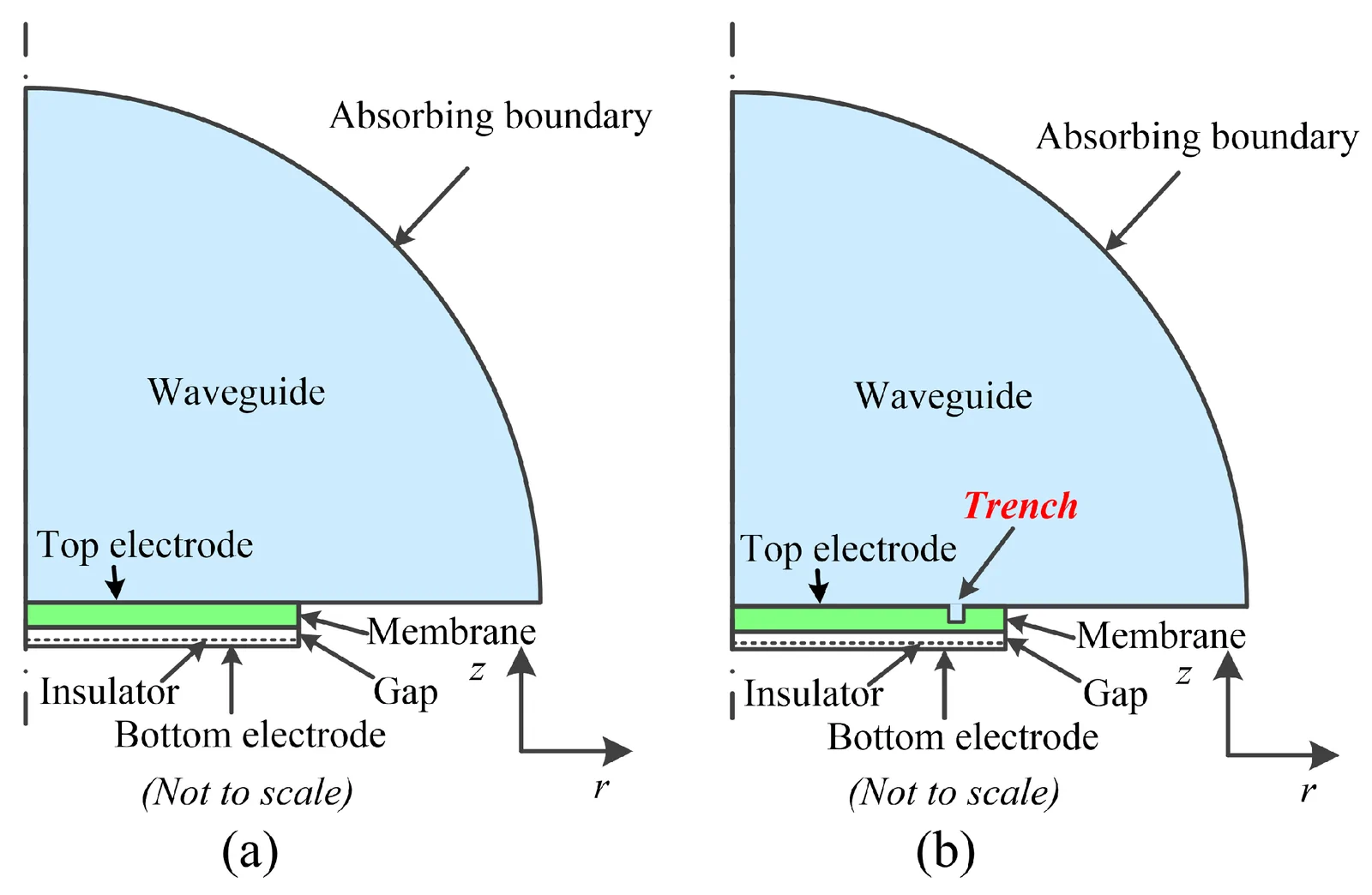
CMUT models developed in COMSOL (**a**) Uniform membrane CMUT; (**b**) trenched membrane CMUT. *r* denotes the radial direction and *z* denotes the vertical direction.

Two two-dimensional axisymmetric FE models of CMUT cells were developed using the FEA software COMSOL 5.3a. (COMSOL, Inc., Stockholm, Sweden). Figure 4a is the uniform membrane CMUT, while Figure 4b is the CMUT with a trenched membrane. Both models share the same dimensions and material properties, which are detailed in Table 2 and Table 3, respectively.

Each CMUT model consists of a CMUT cell and a hemispherical waveguide. The CMUT cell includes bottom electrode, SiO_2_ insulation layer, cavity, low-stress Si_3_N_4_ vibrating membrane, and top electrode structure. The vibrating membrane is made by plasma-enhanced chemical vapor deposition (PECVD) Si_3_N_4_. To achieve high output pressures, both the uniform membrane CMUT and the trenched membrane CMUT utilize full electrode structure [28]. For simplification, the top and bottom electrodes are considered to be infinitely thin. Above the CMUT cell is a hemispherical acoustic waveguide medium, with a height greater than one wavelength. The material of the acoustic waveguide is water, and an acoustic absorbing boundary condition is applied around it to eliminate reflections.

The FE model employs the electromechanical (emi) and pressure acoustic (acpr) acoustics. The emi physics includes the CMUT cell and the acoustic waveguide medium, while the acpr physics includes only the acoustic waveguide. The emi is coupled to the acpr through the “acoustic load per unit area” boundary condition, and the acpr is coupled to the emi via the “normal acceleration” boundary condition.

The prestressed analysis is conducted to investigate the output acoustic pressure of the CMUT in the collapse mode. The DC bias voltage is gradually increased until it exceeds the pull-in voltage, causing the membrane to collapse. Subsequently, an AC voltage with varying frequencies is superimposed to analyze the CMUT’s output acoustic pressure response. To avoid convergence problems with the solver when the membrane contacts the insulation layer after collapsing, a penalty or barrier method is introduced in the FE models [29].

### 2.4. Bayesian Optimization Method

CMUTs are fabricated using MEMS technology, which has relatively high time and economic costs. Therefore, FEA is typically conducted first to analyze the device characteristics and calculate the design parameters. In the case of FEA simulations aimed at structural optimization of CMUTs, multiple iterations are required to find the optimal structure, which can be quite time-consuming. To improve the efficiency of CMUT design, the Bayesian optimization method is employed to the FEA process, aiming to identify the trench parameters that are most effective for enhancing the output acoustic pressure of the CMUT.

Bayesian optimization is a global optimization method based on probabilistic surrogate models, particularly well-suited for black-box optimization problems where the objective function is expensive to evaluate, lacks an explicit analytical expression, or is subject to significant noise [30]. Bayesian optimization leverages historical evaluation data to intelligently select the next sampling point through an active learning strategy, thereby approaching the global optimum with as few function evaluations as possible. The method constructs a surrogate model to approximate the objective function and uses an acquisition function to intelligently select the next sampling point, thereby facilitating the identification of near-optimal solutions with a limited number of evaluations. In this study, Bayesian optimization is utilized to efficiently optimize the trench position and other dimension parameters within a limited computational budget, aiming to maximize output acoustic pressure. The approach follows these steps: First, a Gaussian process (GP) is employed as the surrogate model to fit the acoustic pressure responses from FEA simulations. Next, an acquisition function is introduced to balance global exploration and local exploitation, adaptively selecting the next trench position to be simulated. Finally, the surrogate model is updated iteratively, enabling rapid convergence to the global acoustic pressure extremum with minimal finite-element sampling. In this study, Bayesian optimization is implemented using the built-in Bayesian optimization function bayesopt() in MATLAB R2024b. The specific steps are briefly described as follows.

#### 2.4.1. Define Objective Function

Since bayesopt() in MATLAB only supports the minimization of objective functions, and the optimization goal is to maximize the peak acoustic pressure P(r) of the CMUT, the original maximization problem is equivalently transformed into a minimization problem of the negative acoustic pressure. The optimization formula is shown in (26).

(26) $${r_{2}}^{*}=\arg\min_{r\in [r_{1},r_{2}]}\left\{-P\left(r\right)\right\}$$

where $r_{2}^{*}$ represents the optimal radial arrangement position of the trench obtained through Bayesian optimization, while *r* is the design variable to be optimized, indicating the radial coordinate of the trench. P(r) denotes the peak acoustic pressure from the COMSOL simulation, with larger values being preferable. The objective of Bayesian optimization is to minimize −P(r). The values $r_{1}$ and $r_{2}$ define the range for the radial positions during the trench optimization, with the condition $r_{1}\leq r_{1}^{*}\leq r_{2}$, where $r_{1}^{*}$ is the optimal position estimated theoretically in (25) of Section 2.2.

#### 2.4.2. Select Surrogate Model

GP is employed as the probabilistic surrogate model [31]. GP is a stochastic process defined over the input space, where any finite-dimensional marginal distribution follows a multivariate normal distribution. It is completely characterized by the mean function μ(r) and the covariance function (kernel function) $k(r,r^{\prime })$. The GP prior distribution for the objective function −P(r) is defined as (27):

(27) $$-P\left(r\right)∼\mathrm{GP}(\mu \left(r\right),k\left(r,r^{\prime }\right))$$

where μ(r) is the mean function, which is typically assumed to be a constant to simplify calculations. The function $k(r,r^{\prime })$ is the covariance (kernel) function that measures the similarity between the predicted radial position *r* and the historical sample positions $r^{\prime }$. This study uses the squared exponential kernel as in (28).

(28) $$k\left(r,r^{\prime }\right)=\sigma_{f}^{2}\exp\left(-\frac{{\left(r-r^{\prime }\right)}^{2}}{2l^{2}}\right)$$

Here, $\sigma_{f}^{2}$ represents the signal variance, and *l* denotes the length scale, both of which control the smoothness and fitting range of the surrogate model. These two parameters are optimized from the observed data using maximum likelihood estimation as hyperparameters. Based on the GP prior, the posterior probability distribution of the objective function can be obtained by combining the FE model with the observed simulation data, as shown in (29).

(29) $$-P\left(r\right)|D∼\mathrm{GP}(\mu_{D}\left(r\right),k_{D}\left(r,r^{\prime }\right))$$

where *D* is the existing FEA simulation data, including trench positions and their corresponding negative pressure peak values. $\mu_{D}\left(r\right)$ is the posterior mean function, predicting the negative pressure at a candidate trench position, while $k_{D}\left(r,r^{\prime }\right)$ is the posterior covariance function representing prediction uncertainty. Together, they inform the acquisition function and the selection of the next sampling point.

#### 2.4.3. Choose Acquisition Function

The acquisition function selects the next evaluation point based on the current posterior GP. Expected improvement function is employed as the acquisition function, balancing local exploration near the optimum with global exploration in uncertain areas. Expected improvement is defined as the anticipated enhancement in minimizing the target −P(r) compared to the current best observation, measuring the expected improvement in the objective function value at input point *r* relative to the current optimum, as defined in (30).

(30) $$\alpha_{\mathrm{EI}}\left(r\right)=E\left[\max\left(0,\left(-P_{\mathrm{best}}\right)-\left(-P\left(r\right)\right)\right)\right]$$

where $\alpha_{\mathrm{EI}}\left(r\right)$ represents the expected improvement at point *r*, with E[·] as the mathematical expectation. The function max(0,·) ensures that if the value is negative, it returns 0, indicating no performance improvement at that sampling point. $P_{\mathrm{best}}$ refers to the maximum peak acoustic pressure of the CMUT from FEA simulated samples, corresponding to the historical optimum value $-P_{\mathrm{best}}$ for the minimization target −P(r). Each iteration determines the next sampling position by maximizing the acquisition function. The selection rule, as shown in (31), seeks the next candidate trench position $r_{\mathrm{next}}$ that maximizes $\alpha_{\mathrm{EI}}\left(r\right)$ within the radial constraint $r_{1}\leq r\leq r_{2}$, which serves as input for the next COMSOL FEA simulation. Maximizing $\alpha_{\mathrm{EI}}\left(r\right)$ is equivalent to identifying the trench radial position most likely to further decrease the minimization target −P(r), thereby improving the CMUT peak acoustic pressure.

(31) $$r_{\mathrm{next}}=\arg\max_{r\in [r_{1},r_{2}]}\alpha_{\mathrm{EI}}\left(r\right)$$

#### 2.4.4. Bayesian Optimization Process

**Figure 5:**
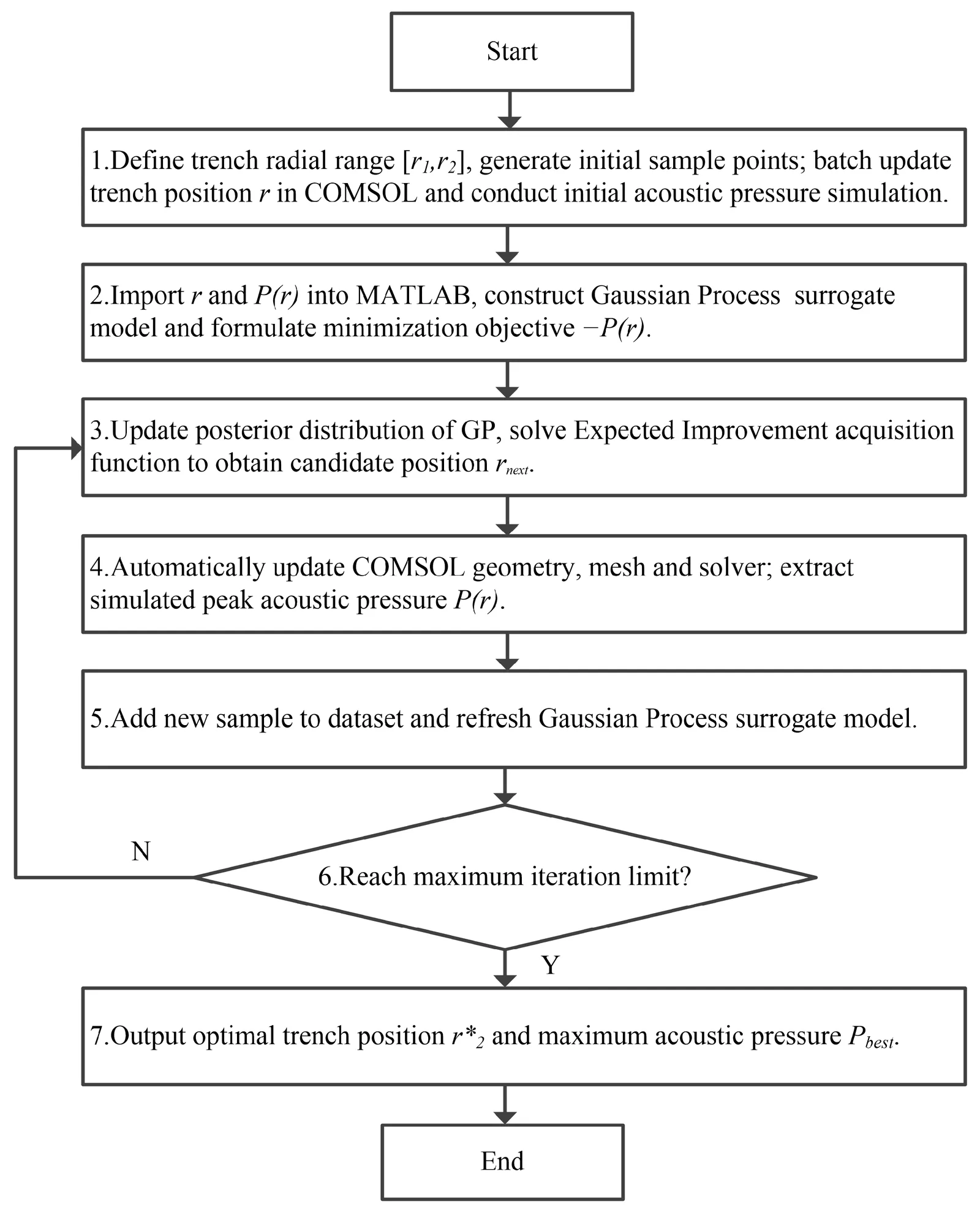
Flowchart of Bayesian optimization for CMUT trench position.

In this study, MATLAB and COMSOL are integrated using LiveLink for MATLAB, and the Bayesian optimization method is utilized to optimize the radial position and dimensions of the trench on the CMUT membrane. Figure 5 illustrates the Bayesian optimization workflow for the trench radial position; the optimization steps for trench depth and width are similar in principle. Regarding the three-parameter global optimization, the radial position, depth, and width are updated concurrently to perform iterative calculations.

1. Define the search space and initialize. The trench position range is set to $\left[r_{1},r_{2}\right]$, centered around the theoretically estimated optimal position $r_{1}^{*}$ from (25) in Section 2.2. Initial sample points are generated, and the corresponding acoustic pressures are obtained via COMSOL simulations.

2. Construct the GP surrogate model. The data pairs (r,P(r)) are imported into MATLAB, where a GP model is constructed. The optimization objective is formulated as minimizing the negative acoustic pressure, namely −P(r).

3. Update the GP and solve the acquisition function. The posterior distribution of the GP is updated, and the expected improvement acquisition function is solved to determine the next candidate trench position $r_{next}$.

4. Run FEA simulation. The COMSOL geometry, mesh, and solver settings are automatically updated (via MATLAB scripting), and the acoustic pressure response is obtained.

5. Update the dataset and GP model. The new sample data is added to the dataset, and the GP surrogate model is refreshed.

6. Check convergence and output. Steps 3–5 are repeated until the maximum number of iterations is reached or the change in acoustic pressure falls below a predefined threshold. The global optimal trench position $r_{2}^{*}$ and the corresponding maximum acoustic pressure $P_{\mathrm{best}}$ are then output.

## 3. Research Method Validation

To validate the effectiveness of the proposed Bayesian optimization-based MATLAB-COMSOL co-simulation optimization method in the design of CMUT output acoustic pressure, we conducted tests on the previously studied embossed pattern CMUT models and devices [22,23]. The mechanism for enhancing the output acoustic pressure in embossed pattern CMUTs is analogous to that of trenched membrane CMUTs, as both approaches rely on optimizing the membrane geometry of collapse-mode CMUTs to increase the vibrational amplitude and thereby improve the output acoustic pressure. In addition, the FE model of the embossed pattern CMUT shares the same modeling framework, boundary conditions, material parameters, and excitation settings as the trenched design, with the only difference being the membrane geometry. The embossed pattern CMUT thus serves as a representative case for validating the optimization method proposed in this study.

### 3.1. FEA Simulation Validation

Similar to this research, the embossed pattern structure also has an optimal position on the annular vibrating membrane of the collapse-mode CMUT that can achieve maximum enhancement of acoustic pressure.

**Figure 6:**
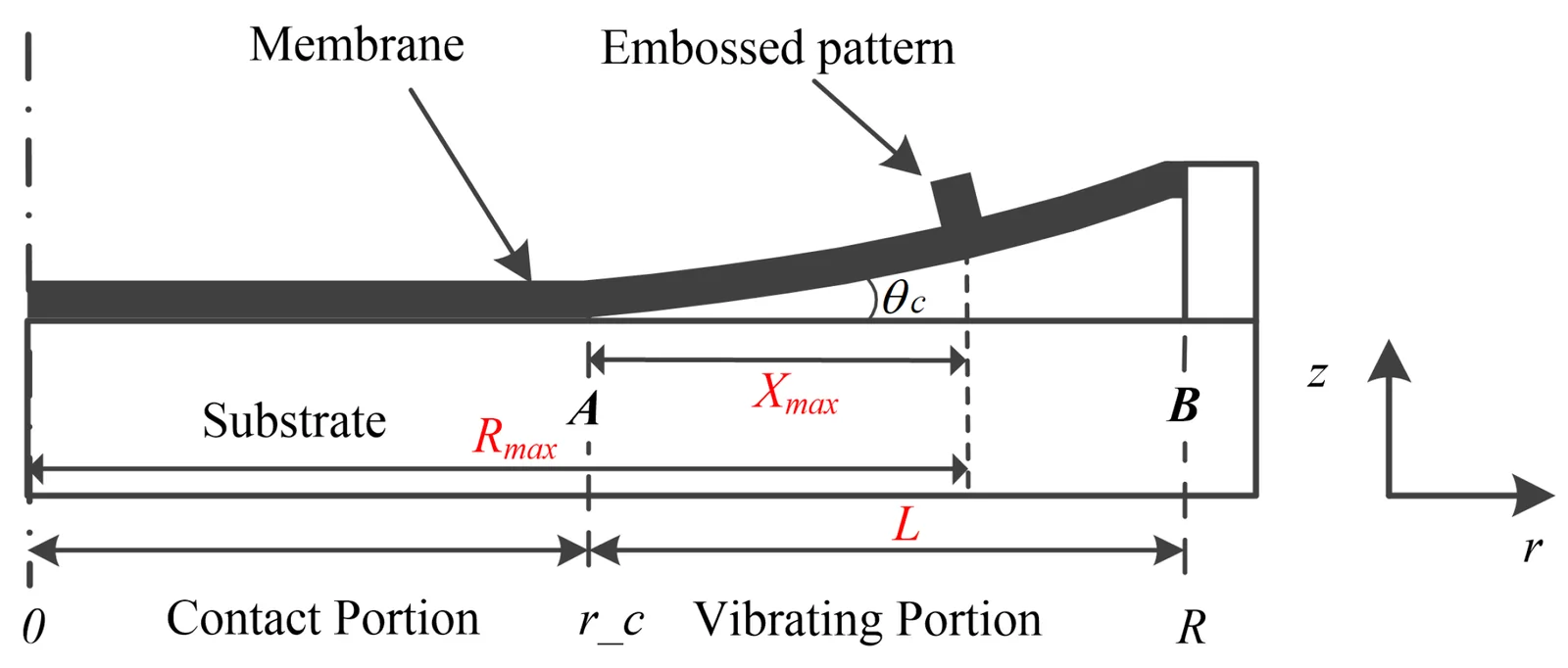
Schematic of the collapse-mode CMUT with an embossed-pattern membrane. *R* is radius of membrane, r_c is the contact radius of membrane, *A* denotes the contact point of membrane on substrate, *B* denotes the edge of membrane on the support, *L* donates the width of the non-contact region, $X_{\max}$ donates the optimal position of the embossed pattern in the non-contact region, and $R_{\max}$ donates the optimal radial position of the embossed pattern.

A schematic of the collapse-mode embossed pattern membrane CMUT is shown in Figure 6. The membrane radius is denoted as *R*, the contact radius as r_c, and the width of the non-collapsed annular region as L=R−r_c. $X_{\max}$ represents the optimal position of the embossed pattern within the non-contact region that maximizes the contribution to output acoustic pressure. The relationship between $X_{\max}$ and *L* is given by [22]:

(32) $$X_{\max}\approx 0.481L.$$

Accordingly, the optimal radial position $R_{\max}$ of the embossed pattern is expressed as

(33) $$R_{\max}=r\text{\_}c\;+X_{\max}$$

The embossed-pattern position was determined through FEA simulations prior to device fabrication. However, due to fabrication tolerances or variations in material properties, the fixed embossed pattern may deviate from the optimal position at the intended operating point. Moreover, in the event that the collapse radius is not directly measurable in the fabricated device, it becomes difficult to identify the exact operating condition. In such cases, the optimal operating point can be identified by adjusting the DC bias voltage, which shifts the collapse radius and consequently the corresponding $X_{\max}$, thereby aligning the fixed embossed pattern with the optimal position.

This is physically enabled by the fact that the collapse radius r_c in a collapse-mode CMUT is controlled by the DC bias voltage, which in turn adjusts the non-collapsed annular width *L*. To interpret this relationship, the relative position $R_{\mathrm{rel}}$ of the embossed pattern at a fixed location $R_{\mathrm{fix}}$ is defined as

(34) $$R_{\mathrm{rel}}=\frac{R_{\mathrm{fix}}-r\text{\_}c}{R-r\text{\_}c}=\frac{R_{\mathrm{fix}}-r\text{\_}c}{L}$$

**Figure 7:**
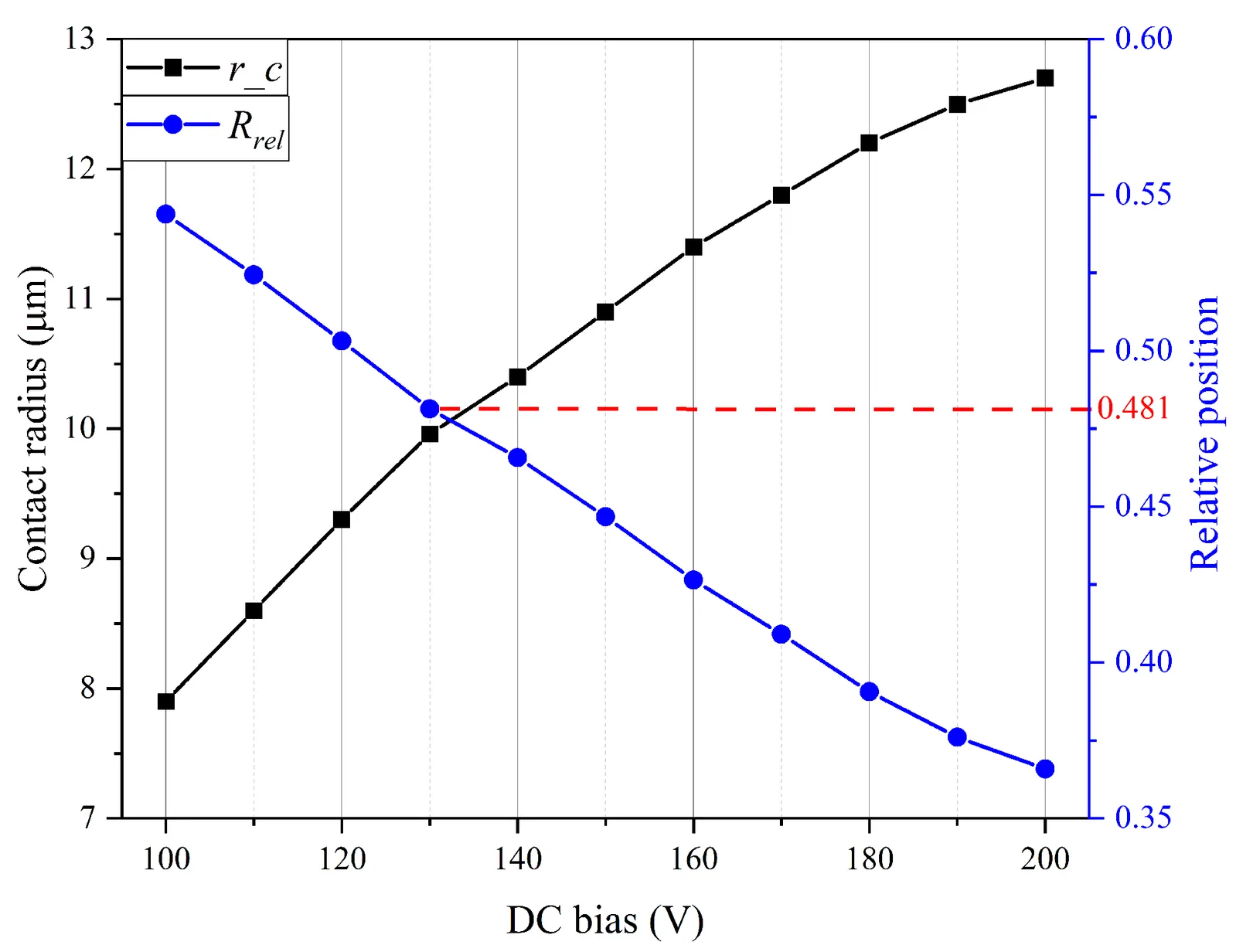
Relationships among DC bias, contact radius and relative position for a fixed embossed pattern. The red dashed line represents the theoretical optimal relative position of 0.481, biased at 130 V.

Figure 7 presents the relationships between $R_{\mathrm{rel}}$, r_c, and the DC bias voltage from FEA simulation. As shown, $R_{\mathrm{rel}}$ and r_c exhibit an inverse relationship. By tuning the DC bias voltage at 130 V, the fixed embossed pattern can be shifted to a relative position of $R_{\mathrm{rel}}\approx 0.481$, thereby satisfying the optimal design criterion of (32). The FE model used in this analysis has different dimensions from the fabricated device; therefore, the specific DC bias voltage required to achieve $R_{\mathrm{rel}}\approx 0.481$ in simulation may differ from that in the experiment. Nevertheless, the underlying physical mechanism, that the collapse radius and thus the optimal embossed-pattern position can be tuned by adjusting the DC bias voltage, remains consistent. Provided that the fixed embossed pattern is not positioned far from the theoretical optimum, this approach can effectively align it with the optimal location by tuning the DC bias voltage to the appropriate level.

**Figure 8:**
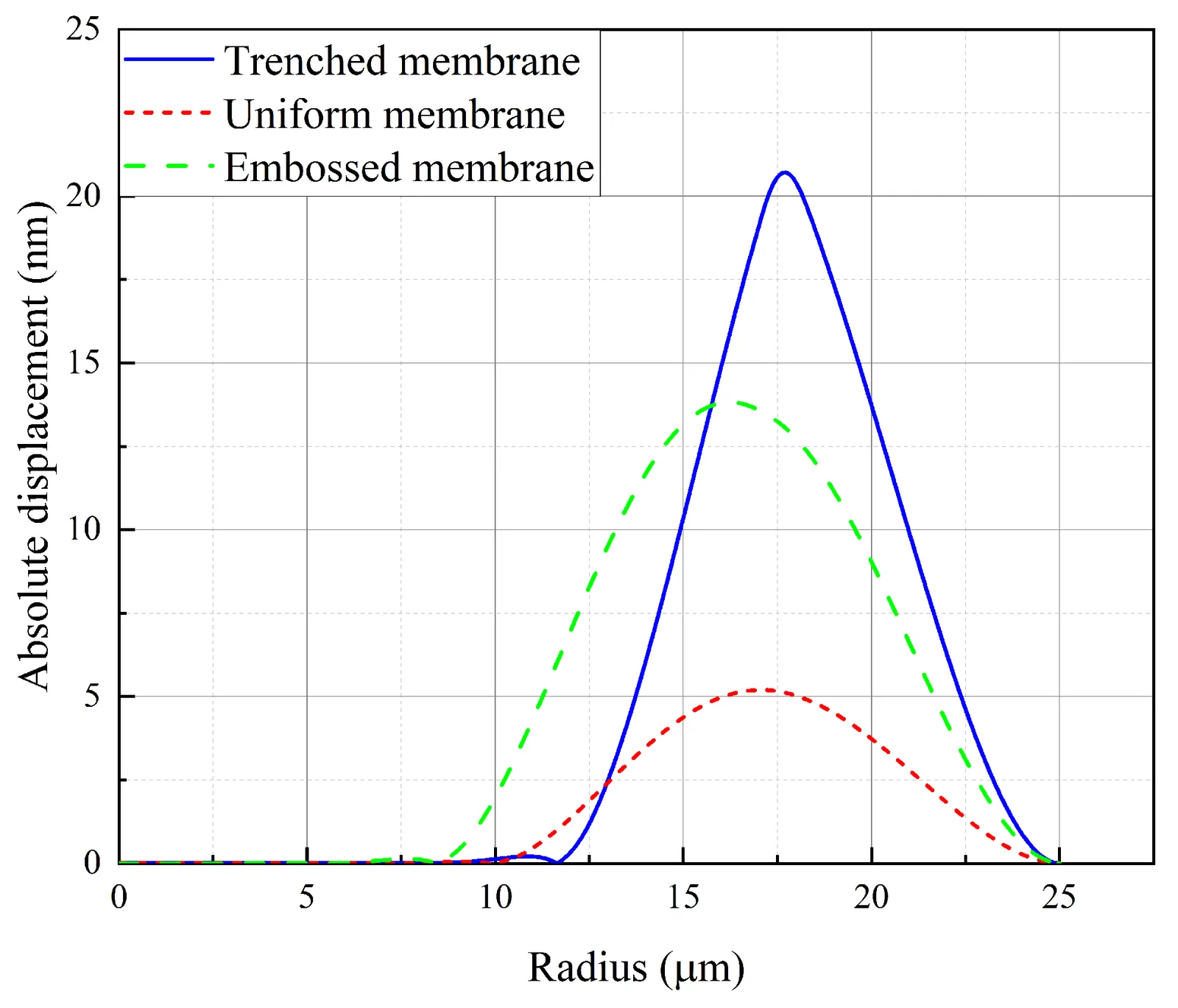
Membrane displacement profiles of trenched, embossed, and uniform CMUTs.

To further validate the simulation results for the trenched design, a comparative FEA study was conducted comparing three configurations: a dimensionally equivalent uniform membrane, the embossed pattern, and the optimized trenched membrane, all under identical excitation conditions at the center frequency. Figure 8 shows the membrane displacement profiles of the three structures. Both the embossed and trenched structures exhibit enhanced piston-like vibration relative to the uniform membrane, consistent with the expected physical behavior. The improved piston-like motion directly contributes to higher output pressure, which is one of the key design objectives of the trenched structure. This comparative study confirms that both structures exhibit physically consistent behavior, supporting the reliability of the FEA predictions for the trenched design.

**Figure 9:**
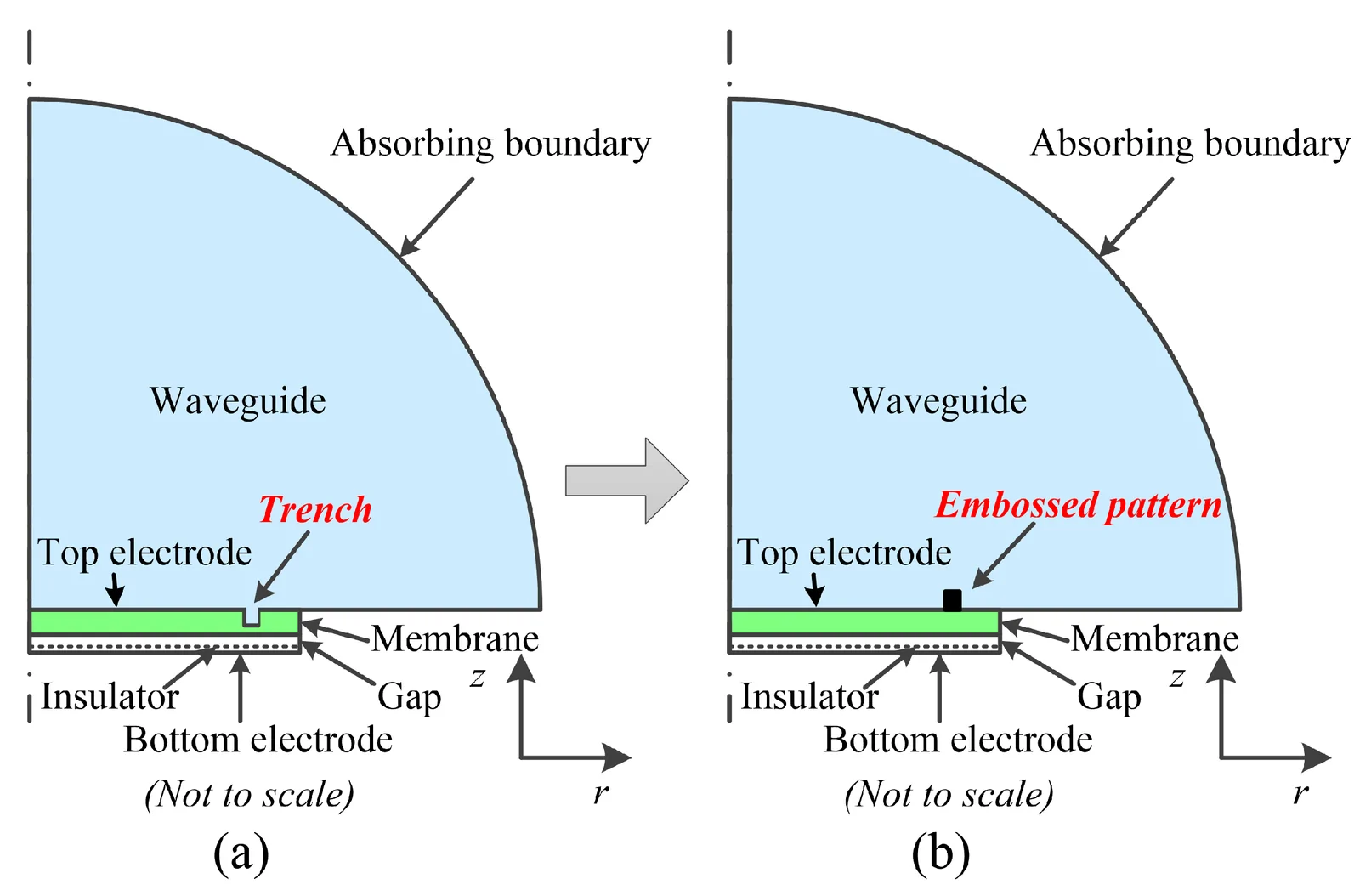
Modified FE models: (**a**) trenched membrane CMUT; (**b**) embossed pattern membrane CMUT.

The modification method for the FE model in COMSOL is illustrated in Figure 9. Specifically, the trench structure membrane in Figure 4b is replaced with an embossed pattern structure membrane, with the dimensions of the embossed pattern being 1 μm × 2 μm. The dimensions, material parameters, and other configurations of the FE model are consistent with those in reference [22]. This ensures that the simulation conditions and results are comparable to those previously established in the reference.

Using the method described in Section 2.4, the range for optimizing the position of the embossed pattern should first be estimated. The membrane radius is 25 μm, and under DC bias voltage of 130 V, the contact radius of the collapsed membrane is 10 μm. That is the width of the uncollapsed annular membrane is 15 μm. According to (33), the theoretically optimal radial position for the embossed pattern $R_{\max}$ is 17.2 μm. Thus, points 1.5 μm to the left and right of this position are selected as the variable range for Bayesian optimization, resulting in an interval of [15.7 μm, 18.7 μm]. The number of optimization iterations is set to 24.

**Figure 10:**
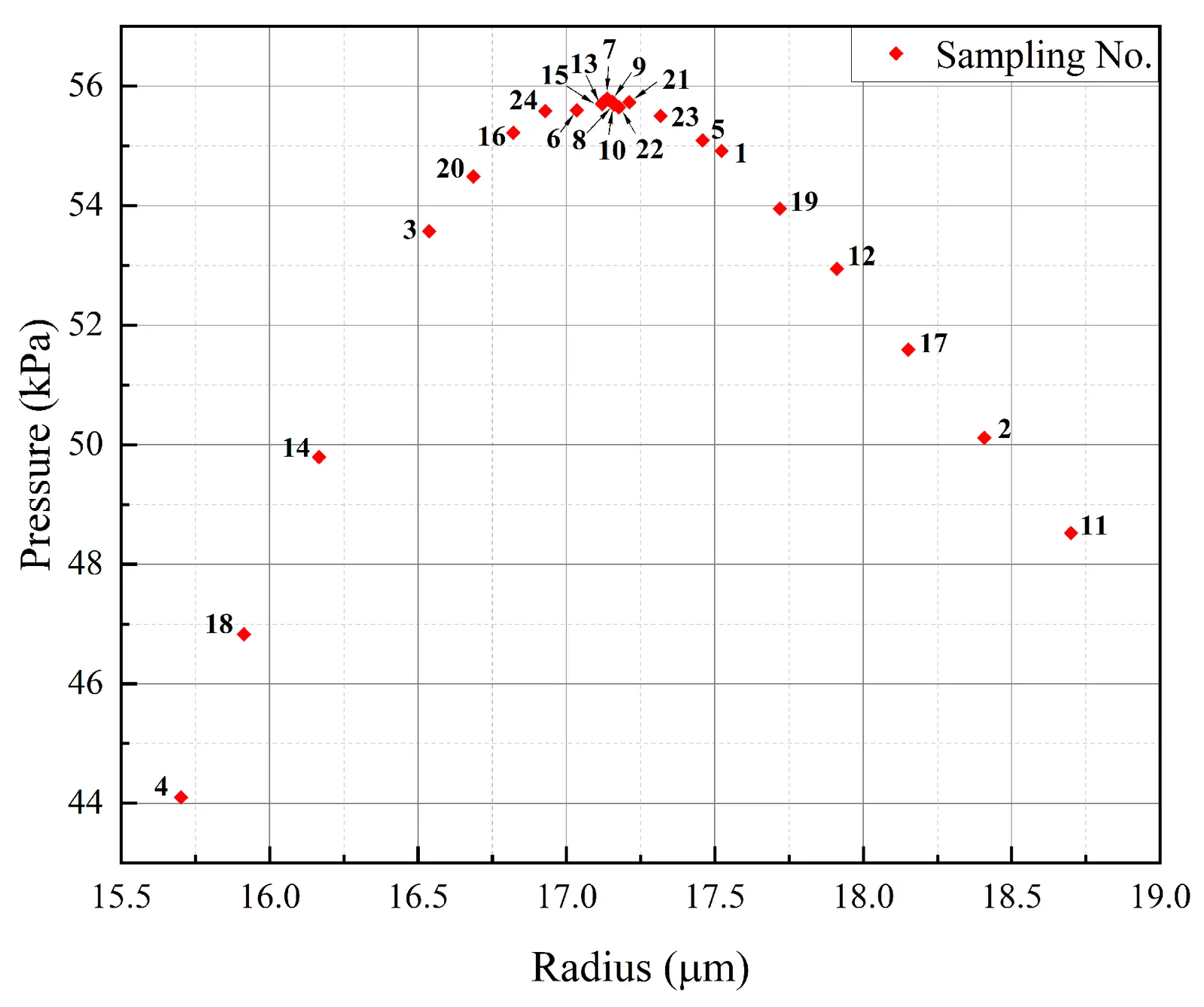
Acoustic pressure vs. embossed pattern radial position for sequential sampling points in Bayesian optimization.

The distribution of sampling points for Bayesian optimization is illustrated in Figure 10. It can be observed from the figure that within the parameter range of [15.7 μm, 18.7 μm], corresponding to the positions of the embossed pattern, the acoustic pressure distribution exhibits a unimodal shape, indicating the presence of a distinct global optimum region. The observation points are distributed relatively uniformly, suggesting that the method has thoroughly explored the parameter space. The position of the embossed pattern that maximizes the increase in acoustic pressure is 17.14 μm, yielding an output acoustic pressure of 55.8 kPa at the 7th sampling point. This position is very close to the optimal value of 17.2 μm found in the FEM simulation from the literature [22], indicating that the Bayesian optimization method used in this study provides a more accurate result.

**Figure 11:**
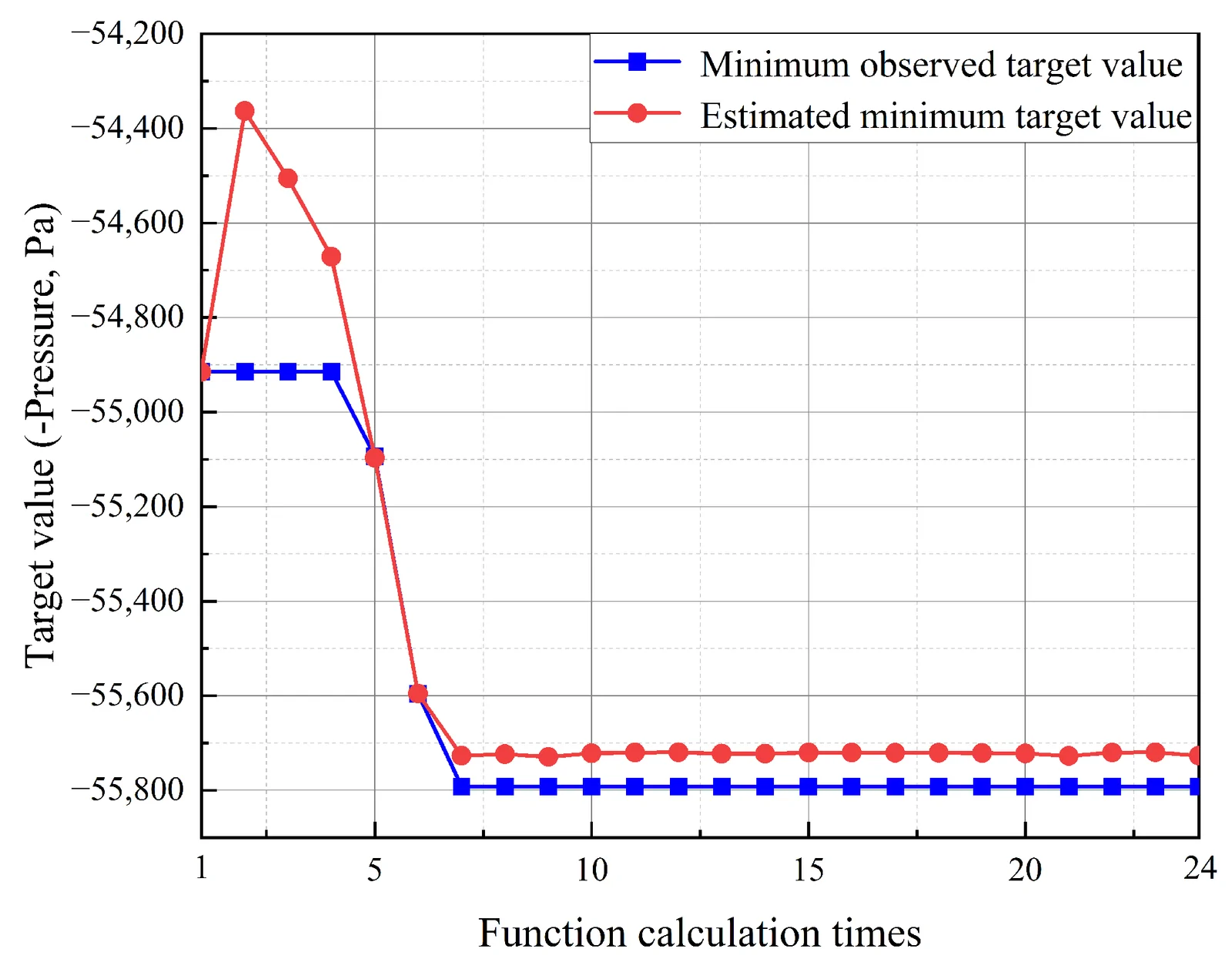
Convergence curve of the Bayesian optimization process for embossed pattern CMUT.

Figure 11 shows the convergence curve of the Bayesian optimization process. The horizontal axis represents the number of completed COMSOL FEA simulations, while the vertical axis indicates the target value (−Pressure, Pa), defined as the negative output acoustic pressure. Smaller values on the vertical axis correspond to higher acoustic pressure. The blue squares denote the observed optimal target values, and the red dotted line represents the globally optimal values predicted by the Bayesian model. Initially, the method constructs a surrogate model through initial sampling, resulting in fluctuating predictions. As iterations progress, between the 5th and 8th simulations, the method quickly identifies the embossed pattern position that significantly enhances acoustic pressure, leading to a substantial decrease in actual target values and refining the surrogate model predictions to align with the observed results. In the later stages, both curves stabilize, indicating that the method has converged to the global optimum. The embossed pattern position at this point represents the optimal arrangement for maximizing the CMUT surface acoustic pressure. This convergence process demonstrates the effectiveness of Bayesian optimization, requiring only about 7 evaluations to identify the optimal solution—less than one-third of the total iterations. This significantly reduces computational costs compared to traditional grid search methods, which often require dozens of evaluations.

The results indicate the advantages of Bayesian optimization in FEA simulation optimization. It can quickly and accurately identify the optimal embossed pattern position for a collapse-mode CMUT to enhance the output acoustic pressure.

### 3.2. Indirect Experimental Validation

**Figure 12:**
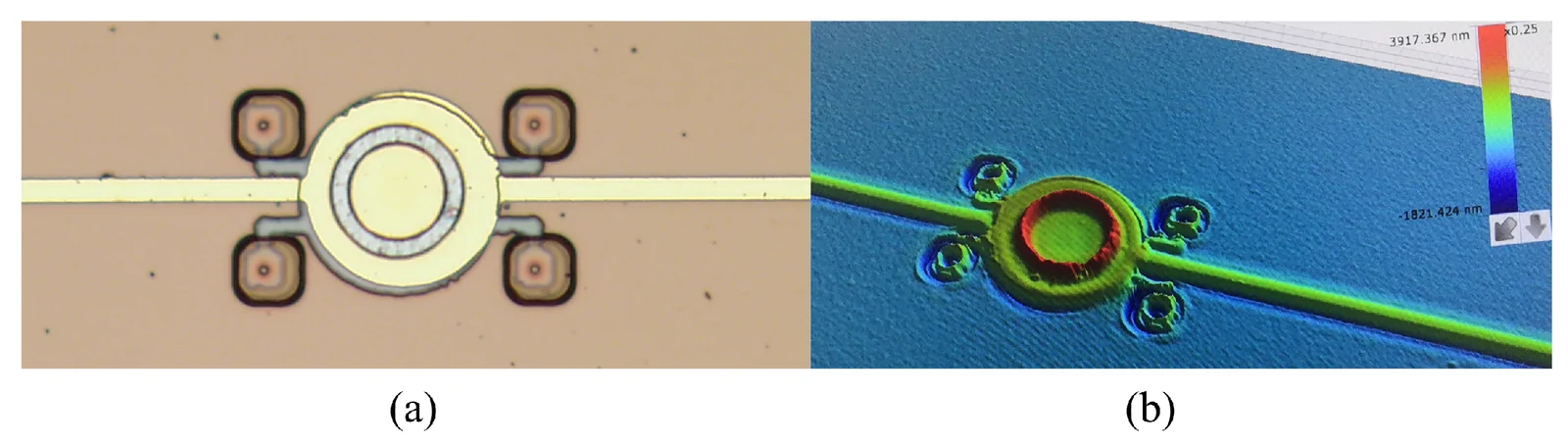
The embossed pattern CMUT (**a**) Optical microscope image; (**b**) three-dimensional surface profile. Note: The negative sign on the axis in (**b**) is generated by the built-in formatting of the three-dimensional optical microscope software.

Furthermore, this method was also indirectly validated on an embossed CMUT cell previously fabricated, shown in Figure 12. The dimensions of the embossed pattern in this CMUT cell are as follows: width is 3 μm, height is 2 μm, inner diameter is 10.5 μm, outer diameter is 13.5 μm, and membrane radius is 20 μm. In reference [23], an FE model was developed to design the embossed CMUT cell, and Bayesian optimization was employed to control the FEA simulation and compared with device experiments. In previous research, although the laboratory equipment could not accurately measure the collapse radius of the CMUT during immersion experiments in collapse-mode, it is possible to adjust the collapse radius by varying the DC bias voltage, thereby aligning the position of the embossed pattern with the vibration center [23].

**Figure 13:**
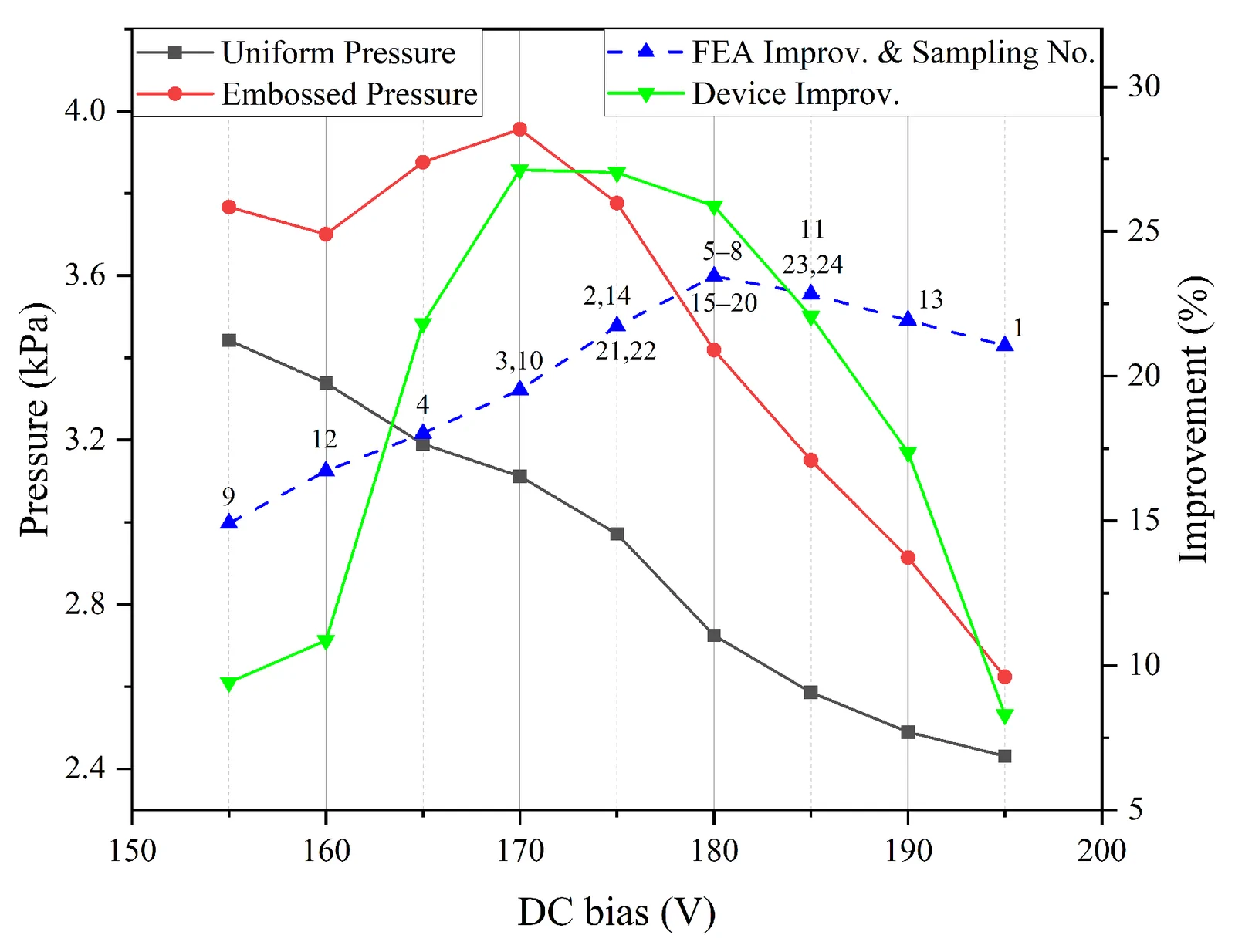
Comparison of the output acoustic pressure and the improvement between Bayesian-optimized FEA and experimental measurements. Dashed line: FEA; solid line: experiment (hydrophone, Ref. [23]). Percentages denote the improvement of the embossed design over the uniform membrane.

In the study, the acoustic pressure improvement of the embossed membrane CMUT compared to the uniform membrane CMUT was evaluated as the objective function for Bayesian optimization, with a total of 24 iterations. Figure 13 shows the output acoustic pressure comparison between FEA sampling points and experimental measurements of the embossed device within the DC bias voltage range of 155 V to 195 V (9 points). The dashed line represents the FEA data, while the solid lines represent the experimental data obtained using a hydrophone (Model: HGL-200, ONDA Co., Sunnyvale, CA, USA), as reported in [23]. The Bayesian optimization method controlled the DC bias voltage of the FE model, iterating in 5 V steps. The numbers next to the FEA observation points in the figure represent the observation points during the Bayesian optimization process. The maximum acoustic pressure enhancement was first identified in the 5th iteration, indicating that the Bayesian optimization method can quickly and accurately determine the optimal DC bias settings to enhance the output acoustic pressure of the collapse-mode CMUT.

Both the simulation and measured curves exhibit a single peak trend, indicating that the optimal position of the embossed pattern is related to the width of the annular vibrating membrane, which also validates the effectiveness of the FE model. The performance of the device declines rapidly after the bias voltage exceeds 180 V, which may be associated with charge trapping and dielectric charging effects in the collapse-mode CMUT [8,32].

In summary, the Bayesian optimization method based on the MATLAB-COMSOL co-simulation framework was indirectly validated using the previously established FE model of the embossed membrane CMUT and the fabricated devices. The experimental results support the effectiveness of the optimization method in identifying bias conditions that enhance output pressure. The observed consistency between the embossed-pattern position and the theoretical optimal criterion further confirms that the optimization workflow is physically consistent with the analytical design criterion. Consequently, this method was applied to the structural design and parameter optimization of the trenched membrane CMUT. Direct experimental validation of the trenched design remains a subject of future work.

## 4. Results

This section details the experimental configuration of the COMSOL and MATLAB co-simulation. The study investigates the single-parameter sequential Bayesian optimization for the radial position, depth, and width of the trench, as well as a global optimization involving all three parameters. The co-simulation was executed on a personal computer equipped with an Intel Core i7-8700 CPU (3.20 GHz) and 32 GB of RAM, utilizing COMSOL Multiphysics 5.3a and MATLAB R2024b as the computational environment, with no parallel computing employed. A typical execution time for one FEA simulation, including geometry reconstruction, mesh generation, and prestressed frequency-domain analysis, required approximately 7 min on average.

### 4.1. Co-Simulation Setup

The FE models described in Figure 4 are used to study output acoustic pressures of uniform membrane and trenched membrane structures of collapse-mode CMUTs. A 130 V DC bias voltage is applied to collapse the membrane, with an 1 V AC voltage is superimposed for frequency sweeping, recording the output acoustic pressure. Under this DC bias, the collapse radius of the membrane was 10 μm, resulting in a ring-shaped vibrating membrane width of 15 μm. Based on (25), the optimal location for the trench was estimated to be 17.5 μm. In this study, initial dimensions of the trench are 1 μm × 0.4 μm in width and depth, respectively.

In Bayesian optimization, bayesopt() function from MATLAB was utilized. In the sequential single-parameter sequential optimization of radial position, depth, and width, the number of initial sample points for each parameter is set to 4, with these points being randomly generated within their respective search boundaries. For the three-parameter global optimization, 18 initial sample points are selected using a Sobol sequence to ensure more uniform coverage across the search space. The stopping criteria of iteration for the single-parameter sequential and three-parameter optimizations are set to 24 and 80, respectively. The GP surrogate model is constructed using the fitrgp function within the bayesopt() framework, employing the default ARD Matérn 5/2 kernel as the covariance function. This kernel is a widely accepted choice in Bayesian optimization, as it is well-suited for characterizing potentially non-smooth responses derived from numerical simulations. Because the simulation process of COMSOL involves mesh generation and numerical solution, its results inherently contain numerical noise. Therefore, the IsObjectiveDeterministic parameter was set at the default value of false in MATLAB. This allows the GP surrogate model to treat the observed objective function values as samples with noise, thereby more robustly handling numerical uncertainty in the simulation. The parameters for the Bayesian optimization are summarized in Table 4.

### 4.2. Single-Parameter Sequential Optimization

#### 4.2.1. Radial Position Optimization

Using the theoretically estimated optimal position of 17.5 μm as the center, the parameter optimization interval was defined as [16 μm, 19 μm]. The Bayesian optimization method was employed with output acoustic pressure as the objective function to search for the optimal radial position of the trench within this interval. A total of 24 sampling iterations were set for the optimization process.

**Figure 14:**
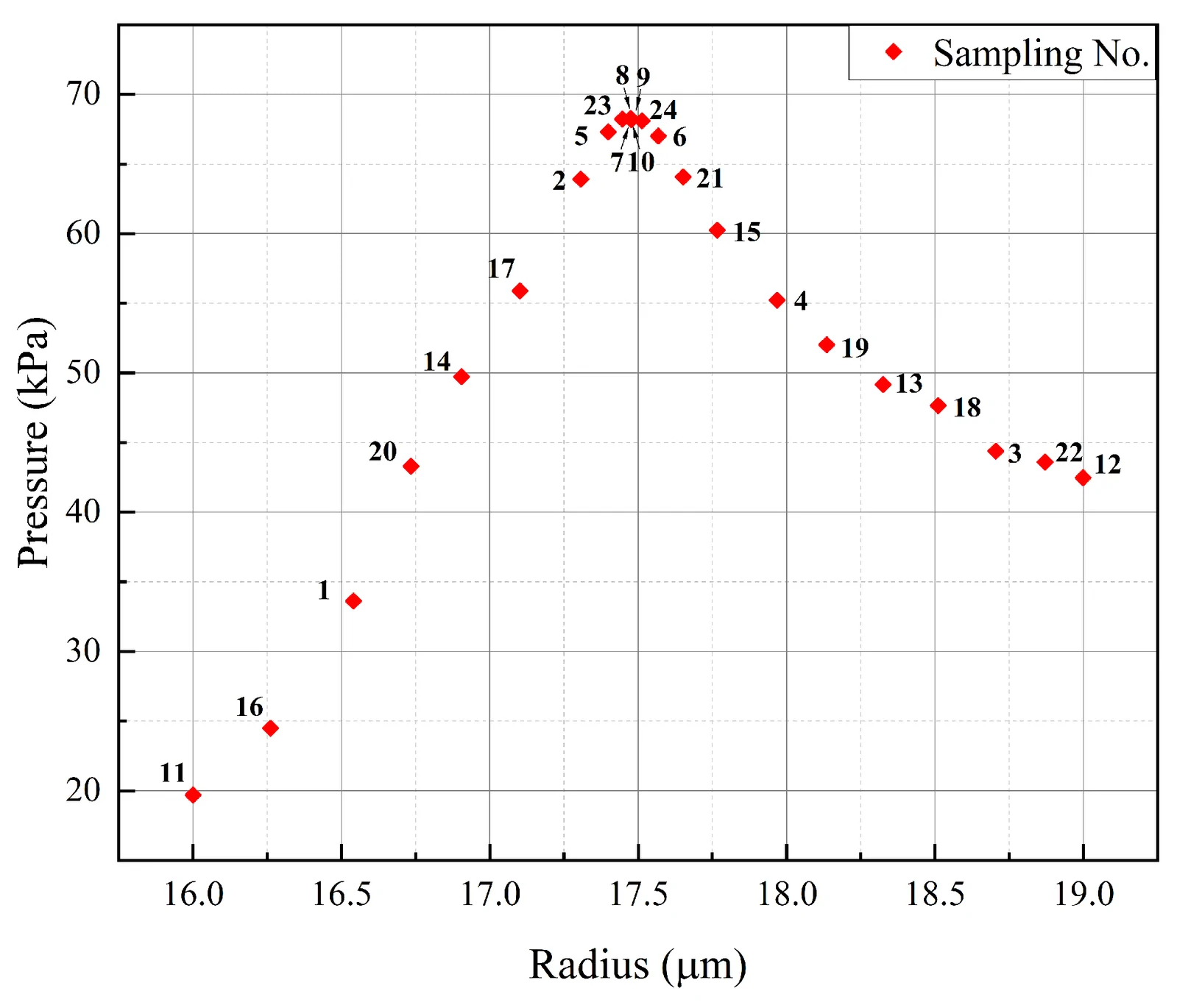
Acoustic pressure vs. trench radial position for sequential sampling points in Bayesian optimization.

Figure 14 demonstrates the trench center position identified by the Bayesian optimization method that yields the maximum output acoustic pressure. The single-peak characteristic indicates the existence of an optimal position for the trench structure on the vibrating membrane. The maximum point corresponds to the data from the 8th sampling, with an output acoustic pressure of 68.28 kPa at a location of 17.48 μm, indicating that this position is the optimal radial position. Under the same conditions, the maximum output acoustic pressure of a uniform membrane CMUT is 36.14 kPa. The output acoustic pressure improvement of the trench-structured CMUT is 88.90%. This prominent performance improvement demonstrates the effectiveness of the trench design for enhancing the acoustic pressure of the CMUT.

**Figure 15:**
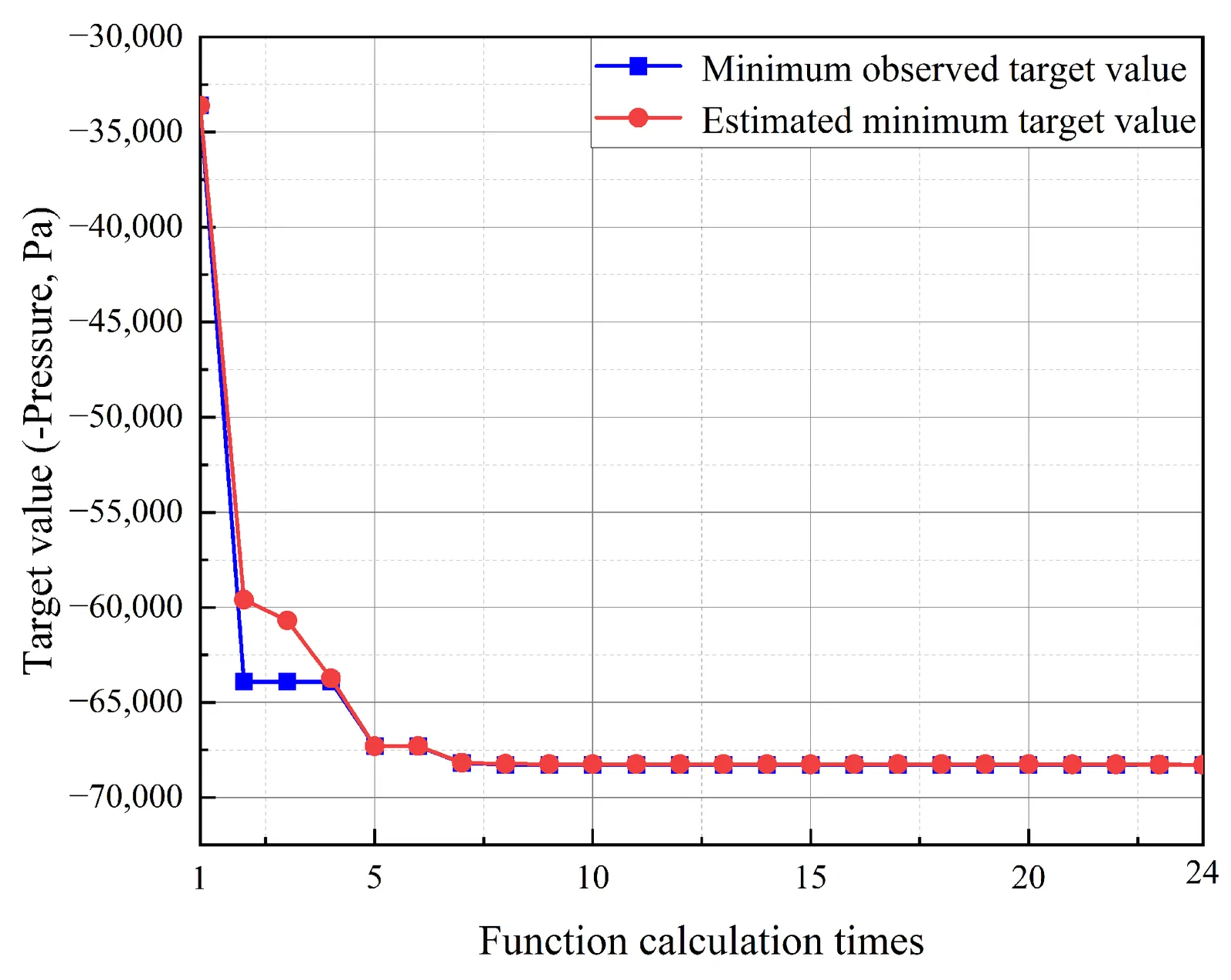
Convergence curve of the Bayesian optimization process for trench radial position.

Figure 15 shows the convergence process of Bayesian optimization for the optimal radial position of the trench. This method quickly located the parameter space that significantly enhances the CMUT acoustic pressure performance within the first few iterations, completing convergence after 8 iterations to find the global optimal solution. It indicates that Bayesian optimization achieved the optimal solution in only one-third of the set iteration count, demonstrating the superiority of this method.

**Figure 16:**
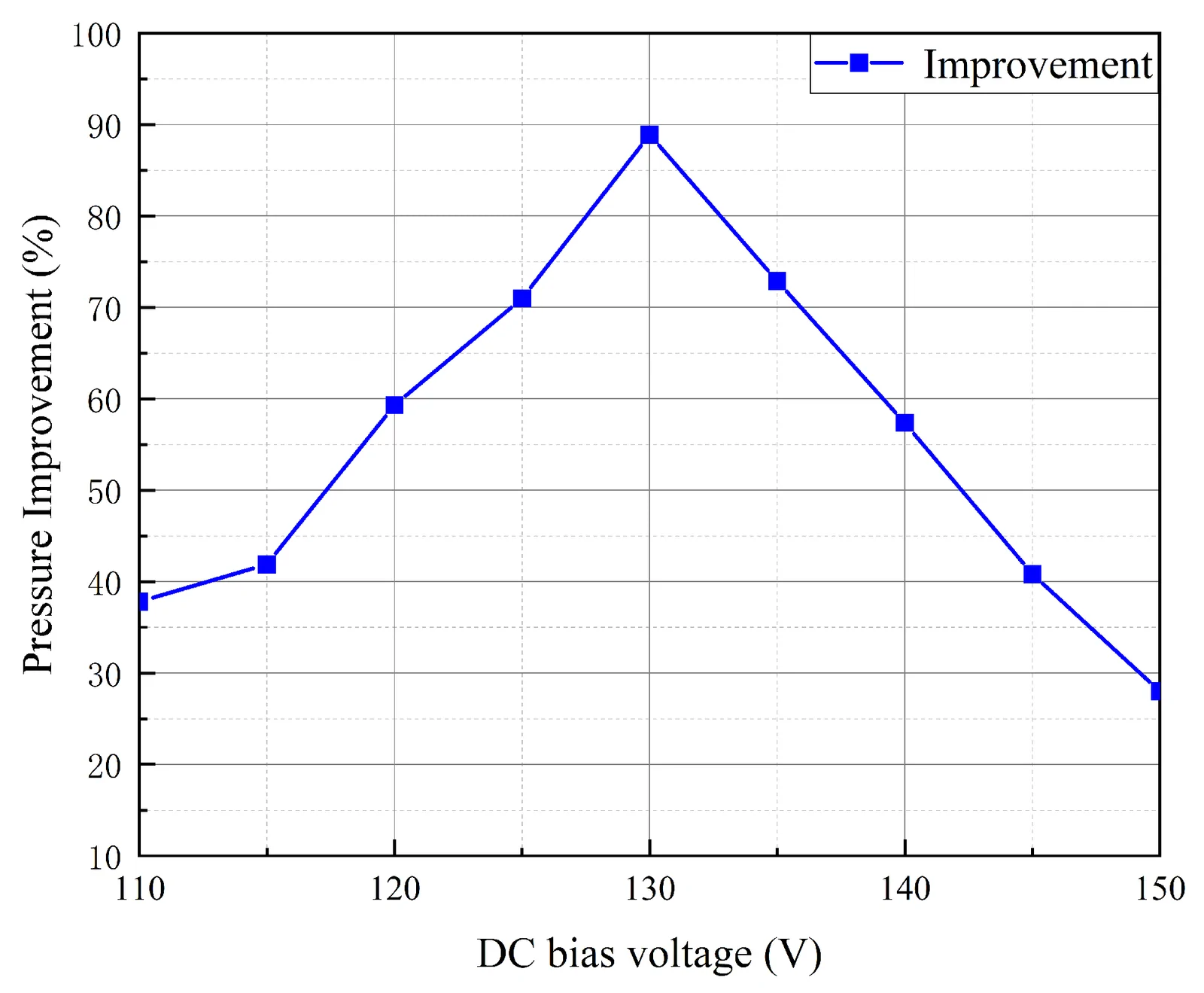
Pressure improvement of collapse-mode CMUT with optimally positioned trench at 17.48 μm under varying DC bias voltages.

Figure 16 is the variation curve of output acoustic pressure improvement of the trenched membrane CMUT with a trench center position of 17.48 μm relative to the uniform membrane CMUT at different bias voltages [110 V, 150 V]. The curve exhibits a single-peak characteristic. The reason for this is that the DC bias voltage in the collapse-mode alters the width of the annular vibrating membrane, effectively adjusting the relative position of the trench structure to the annular membrane. This indicates that for membranes of varying widths, the position of the trench is a key factor in improving output acoustic pressure. Twelve independent Bayesian optimization runs were conducted for the radial position. A width of 17.46 μm, which yielded a maximum acoustic pressure of 68.52 kPa, was selected as the optimal width.

#### 4.2.2. Depth Optimization

**Figure 17:**
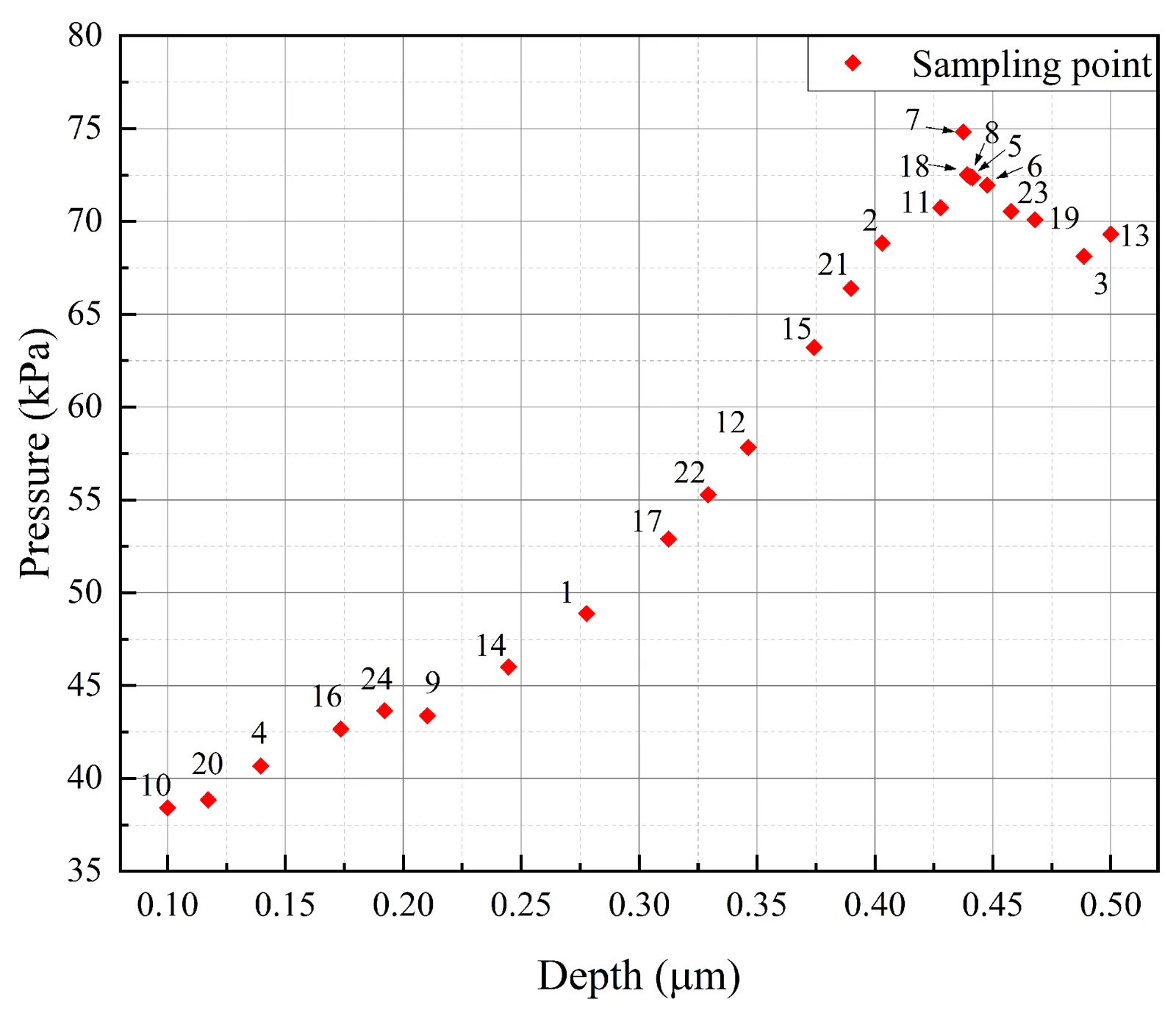
Acoustic pressure vs. trench depth for sequential sampling points in Bayesian optimization.

Based on the findings in Section 4.2.1, the central position of the trench is fixed at 17.46 μm, the trench width is set to 1 μm, and its depth is constrained within the range of [0.1 μm, 0.5 μm]. Bayesian optimization is adopted to maximize the output acoustic pressure of the trenched membrane CMUT by optimizing the trench width, with a total of 24 sampling iterations. The relationship between different FEA sampling points and trench depth is shown in Figure 17. As the trench depth increases from 0.1 μm to 0.42 μm, the acoustic pressure exhibits a monotonically increasing trend. This can be attributed to the fact that, as indicated in (8), the trench reduces bending stiffness through localized thinning, making the vibrating membrane more susceptible to dynamic deformation. However, when the trench depth approaches 0.43 μm, the acoustic pressure distribution shows irregular jumps, with a step peak occurring around 0.44 μm at the 7th sampling point, followed by a decay with further increases in depth. This phenomenon indicates that the system has exceeded the applicability of the simple linear stiffness reduction model proposed in this study. The reason is that when the trench depth exceeds a certain proportion of the membrane thickness, significant geometric discontinuities are formed at the bottom and side walls of the trench, leading to a sharp increase in the stress concentration factor in that region. The system transitions from linear plate behavior to a von Kármán geometric large-deflection state [33], which manifests as jumps and subsequent decay in output acoustic pressure. Therefore, the optimization of trench depth must balance the acoustic pressure gain resulting from stiffness reduction against the risk of nonlinear effects. Considering the fabrication process variations associated with dry etching, the safe threshold for trench depth in this study is set at ≤0.41 μm.

**Figure 18:**
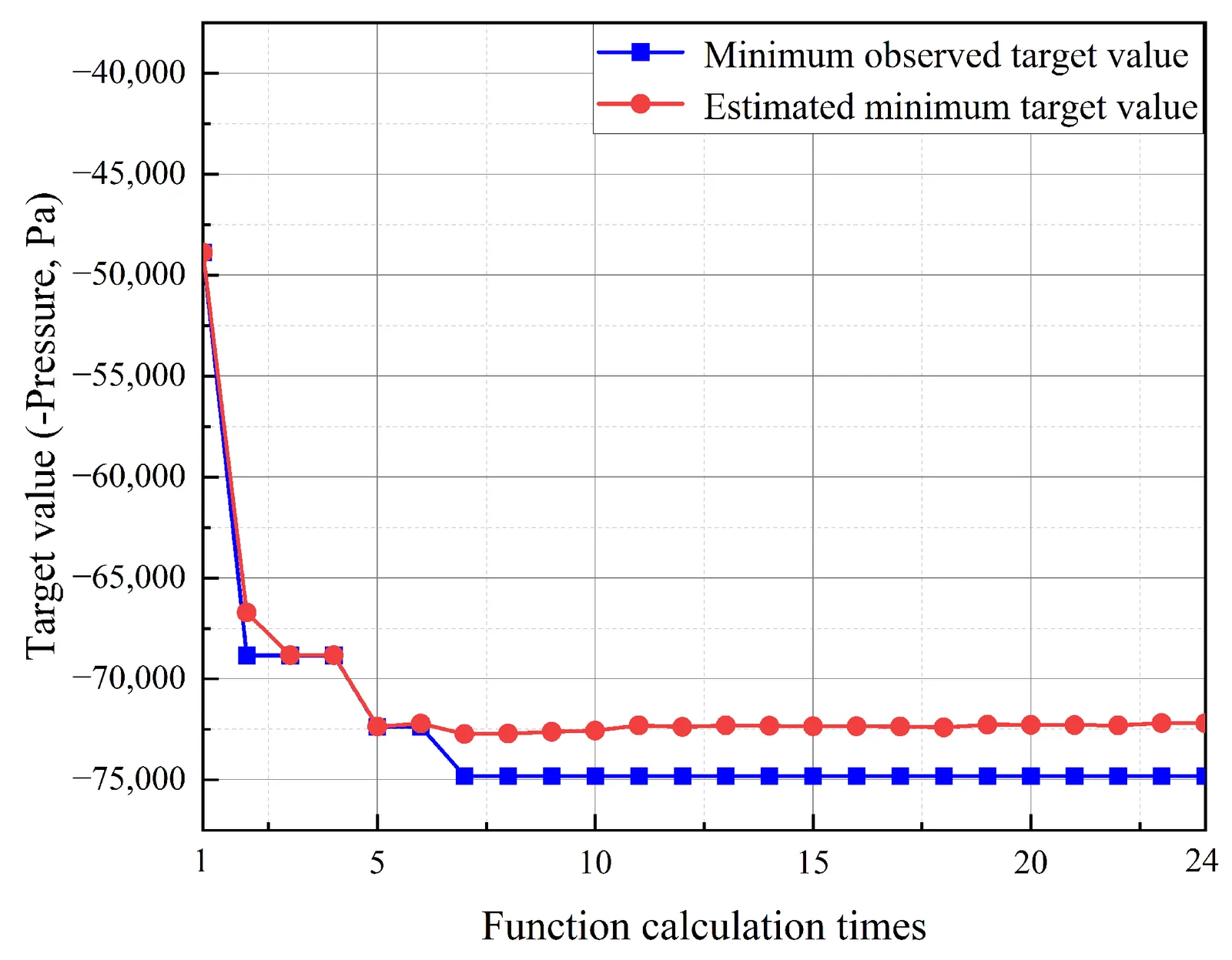
Convergence curve of the Bayesian optimization process for trench depth.

Figure 18 presents the convergence curve of the Bayesian optimization for trench depth, revealing that the global optimal solution was reached after the 7th iteration. This indicates that Bayesian optimization achieved the optimal solution using less than one-third of the total iteration count. The discrepancy between the observed and estimated data after convergence arises from an acoustic pressure jump triggered by membrane nonlinearity at excessive trench depths.

#### 4.2.3. Width Optimization

**Figure 19:**
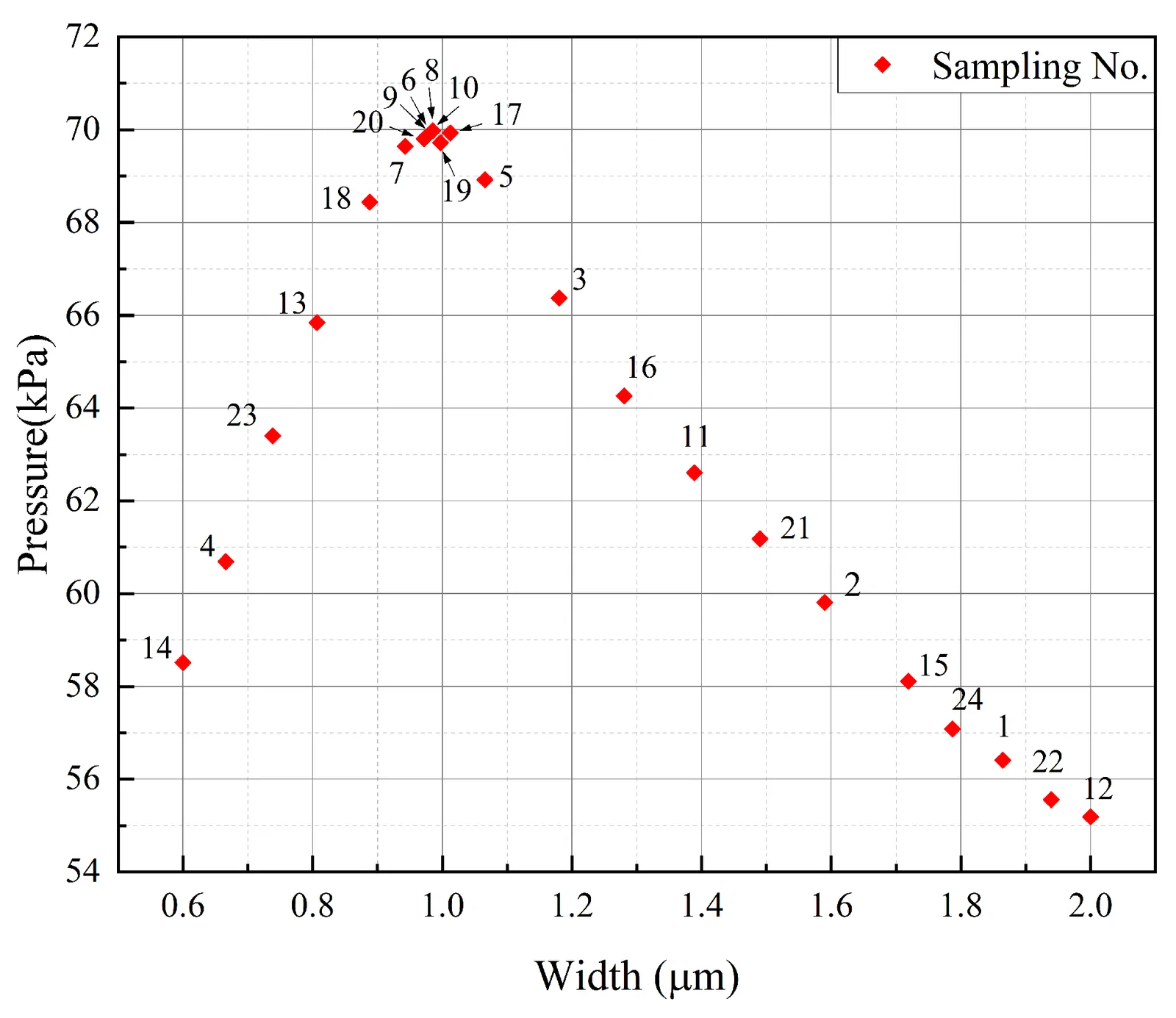
Acoustic pressure vs. trench width for sequential sampling points in Bayesian optimization.

Based on the findings in Section 4.2.1 and Section 4.2.2, the central position of the trench is fixed at 17.46 μm, and the trench depth is set at 0.41 μm. Bayesian optimization is adopted to maximize the output acoustic pressure of the trenched CMUT by optimizing the trench width in the range of [0.6 μm, 2.0 μm], with a total of 24 sampling iterations. Figure 19 shows the distribution of maximum output acoustic pressure collected at different widths. It is evident that the output acoustic pressure exhibits a unimodal characteristic as the width varies. The maximum output acoustic pressure of 69.97 kPa is attained at the 8th sampling point when the trench width is 0.99 μm.

**Figure 20:**
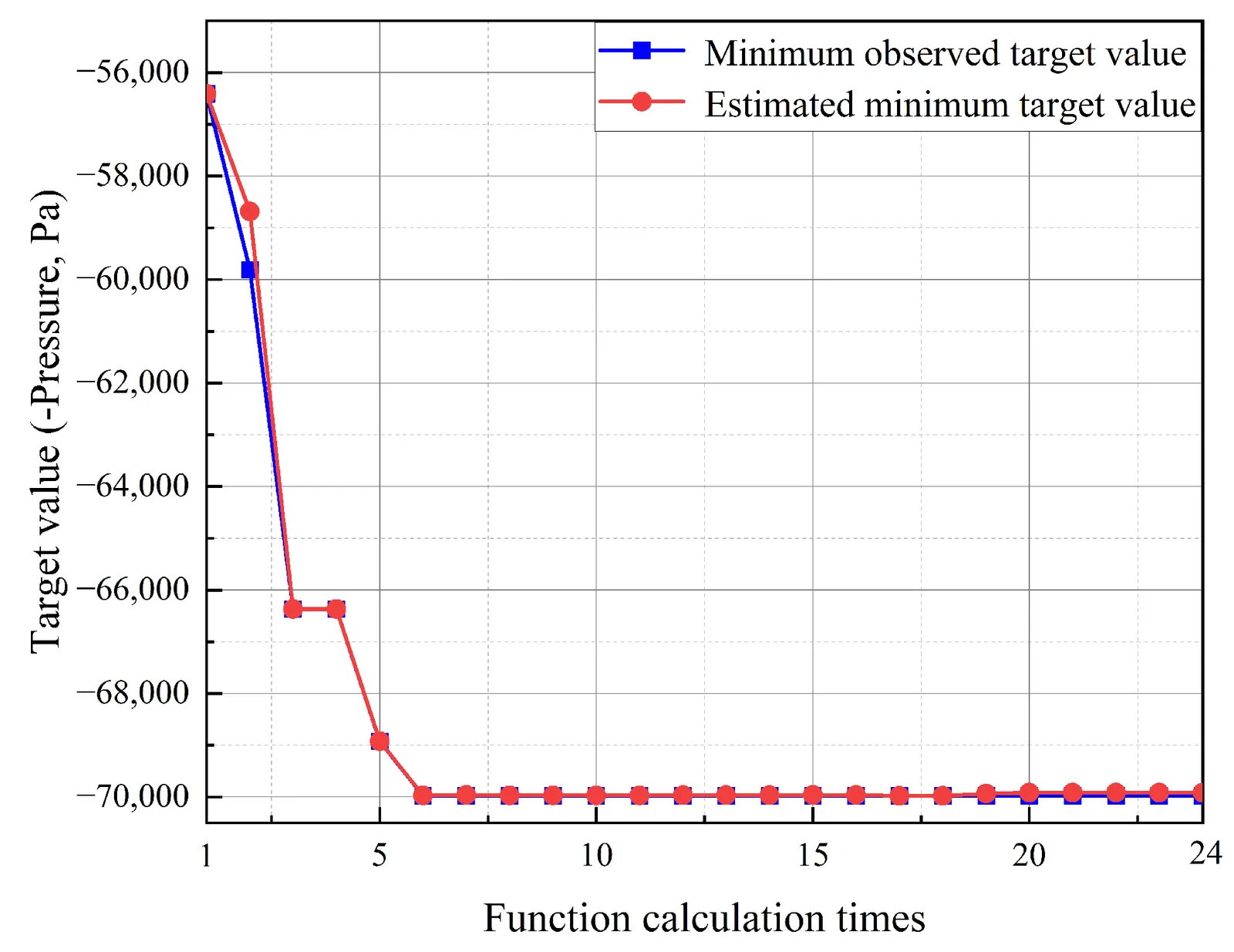
Convergence curve of the Bayesian optimization process for trench width.

Figure 20 presents the convergence curve of Bayesian optimization for the optimal trench width. Initially, the method explored the parameter space, causing deviations between predicted and observed values. As sampling increases, the uncertainty of the GP model decreases, leading to the convergence of the curves. The broad optimization range and the concentration of optimal acoustic pressure around 0.99 μm contribute to high initial uncertainty. However, as sampling continued, the model improved its fitting accuracy, with the curves overlapping and converging after about the 8th iteration. This indicates that Bayesian optimization can find the optimal solution using one-third of the total iterations, demonstrating its efficiency.

Twelve independent Bayesian optimization trials were conducted for the trench width, from which an optimal value of 0.98 μm was identified. With the sequential optimization of all individual trench parameters completed, a peak output acoustic pressure of 70.02 kPa was achieved. This result was obtained at a radial position of 17.46 μm, a depth of 0.41 μm, and a width of 0.98 μm, representing a 93.73% increase over the 36.14 kPa generated by a uniform membrane CMUT in collapse-mode.

#### 4.2.4. Repeated Runs and Stability of Single-Parameter Sequential Optimization

To ensure the reliability and reproducibility of the results, the single-parameter sequential optimizations were repeated 12 times independently. The comprehensive statistical analysis, mean values, standard deviations (SD), CV, as well as minimum, median, and maximum values, is presented in Table 5. Additionally, iteration convergence plots for each parameter have been provided to demonstrate stability.

**Figure 21:**
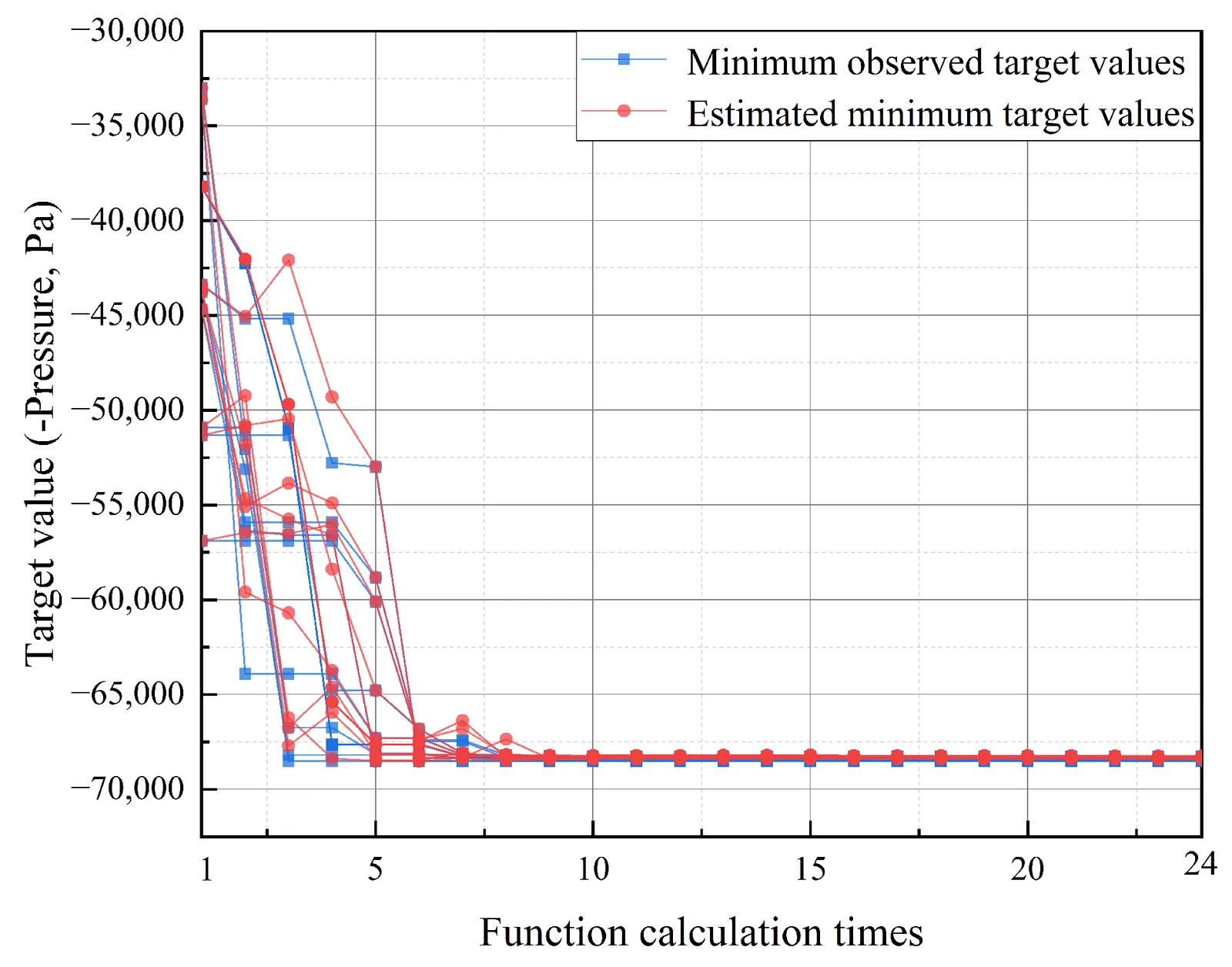
Radial position convergence profiles across 12 independent Bayesian optimization trials.

The optimal radial position remained nearly identical across all runs, ranging from 17.46 to 17.49 μm, with a CV of only 0.05417%. Similarly, the acoustic pressure exhibited high stability, with a CV of 0.142% and a variation range of approximately 236 Pa. These results indicate that the optimal value for the radial position is highly consistent across different random initial seeds, demonstrating excellent optimization reproducibility. Figure 21 illustrates the convergence curves for 12 independent Bayesian optimization runs targeting the trench’s radial position. For the single-parameter sequential Bayesian optimization under varying random initial conditions. The optimization processes typically converge within 3 to 10 iterations, after which the 12 trajectories nearly overlap, with a maximum difference in acoustic pressure of less than approximately 236 Pa. These results demonstrate the high convergence stability of the Bayesian framework; the algorithm consistently and reliably reaches a similar optimal solution within approximately 10 evaluations, regardless of the random initial conditions.

**Figure 22:**
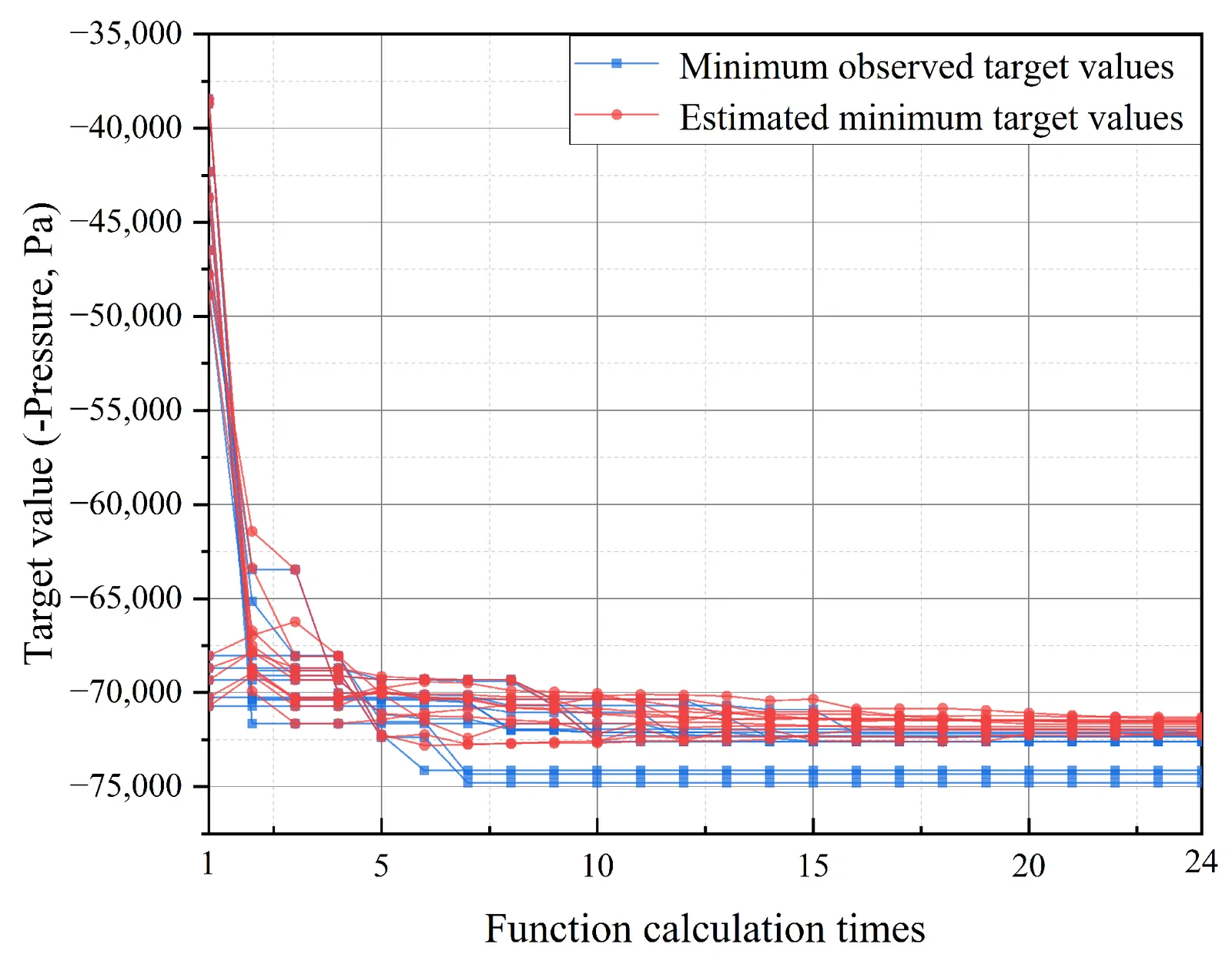
Depth convergence profiles across 12 independent Bayesian optimization trials.

The statistical characteristics of the optimal trench depth and the corresponding output acoustic pressure in Table 5. The CV for the depth is 0.762%, while the CV for the acoustic pressure is 1.344%, with a variation range reachingapproximately 2852 Pa. These elevated CVs are attributed to the transition into a large-deflection regime when the trench depth exceeds 0.43 μm, which triggers nonlinear jumps in acoustic pressure. Consequently, although Bayesian optimization identifies 0.44 μm as the numerical optimum, the maximum trench depth in this study is constrained to 0.41 μm. This constraint maintains linearity and provides a necessary safety margin against unstable vibration regimes. Figure 22 displays the convergence curves for 12 independent Bayesian optimization runs targeting the trench depth, with convergence occurring within 6 to 16 iterations. Compared to the radial position, these curves exhibit a higher degree of dispersion, with pressure differences reaching up to approximately 2852 Pa. This significant spread reflects the stronger nonlinearity of the depth parameter within the parameter space and its high sensitivity to random initial conditions.

**Figure 23:**
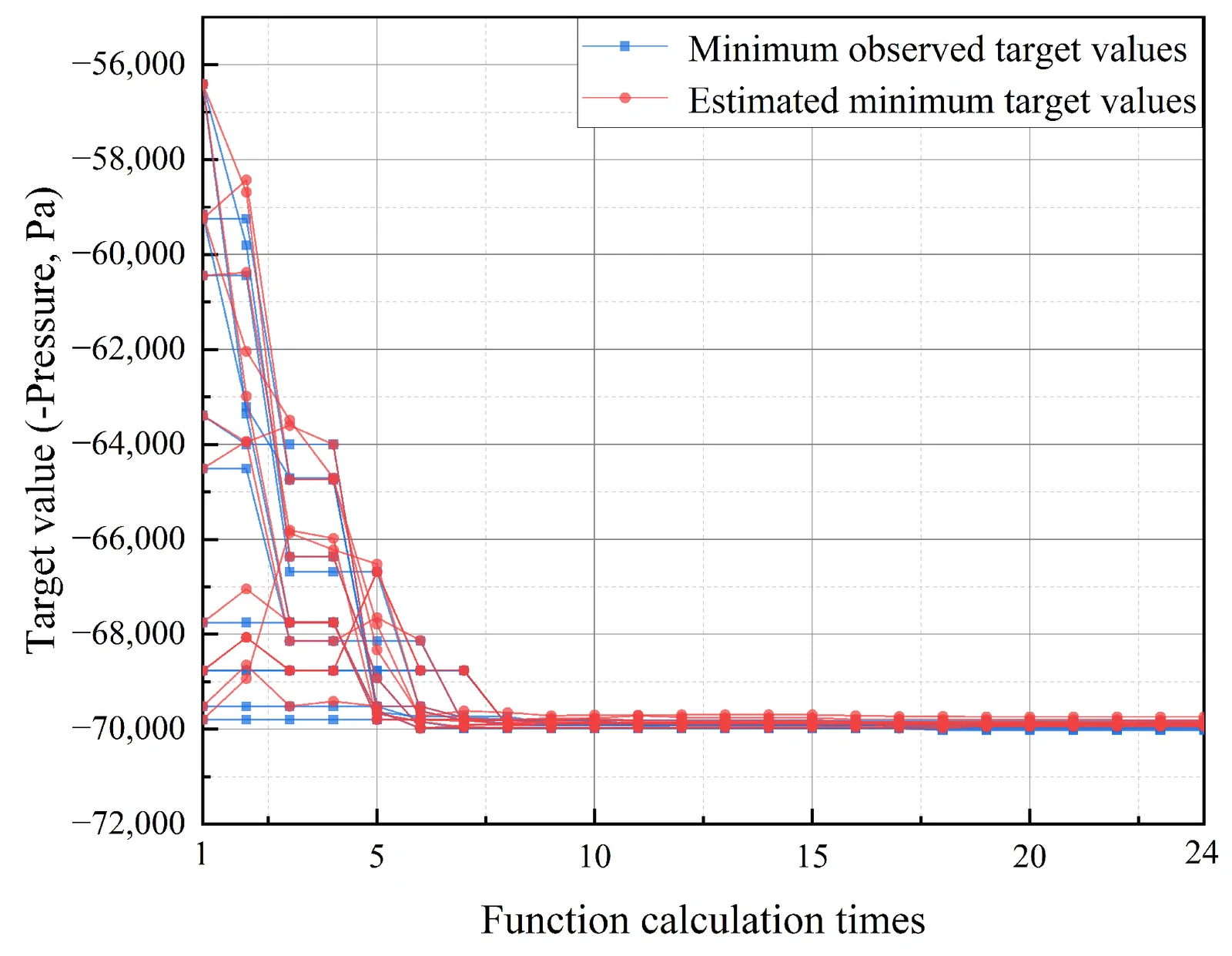
Width convergence profiles across 12 independent Bayesian optimization trials.

In Table 5, although the CV of the width parameter is relatively high (1.654%), the corresponding CV for the acoustic pressure is merely 0.078%, with a variation range of approximately 192 Pa.This indicates the existence of a flat response region in acoustic pressure near the optimal width, suggesting a high fabrication tolerance for the fabrication process. Figure 23 illustrates the convergence curves of 12 independent Bayesian optimization runs for the trench width, all of which achieve convergence within 8 to 18 iterations. The curves exhibit high consistency, with a final spread of less than 200 Pa. This convergence behavior suggests a flat response region near the optimal width. Specifically, despite differing initial sampling points and optimization trajectories, each run yields nearly identical final acoustic pressure levels, demonstrating the lowest sensitivity among the three parameters investigated.

### 4.3. Three-Parameter Global Optimization of Trench Parameters

The preceding sequential analysis of the radial position, depth, and width reveals their respective influences on the output acoustic pressure. These single-parameter sequential optimizations served as a preliminary screening step to identify the approximate optimal range of each parameter. However, since the three geometric parameters may be coupled, the sequential single-parameter sequential approach cannot fully capture their interactions. To comprehensively evaluate the synergistic effects among these variables, a three-parameter global optimization is conducted using the Bayesian framework, in which all three parameters are optimized simultaneously within each iteration.

#### 4.3.1. Optimization Setup and Sampling Distribution

The search space for the global optimization was defined as: radial position [16 μm, 19 μm], depth [0.1 μm, 0.41 μm], and width [0.6 μm, 2.0 μm]. To ensure a comprehensive exploration, each Bayesian optimization run consisted of 80 iterations. Specifically, the process was initialized with 18 sample points generated via a Sobol sequence to achieve a uniform distribution across the three-parameter space. This quasi-random sampling strategy was employed to avoid sample clustering or coverage gaps, thereby facilitating faster convergence toward the global optimum [34,35]. The remaining 62 evaluations were guided by the expected improvement acquisition function. To ensure the statistical robustness and reproducibility of the results, 12 independent trials of this three-parameter Bayesian optimization were conducted, yielding a total of 960 sampling points for analysis.

**Figure 24:**
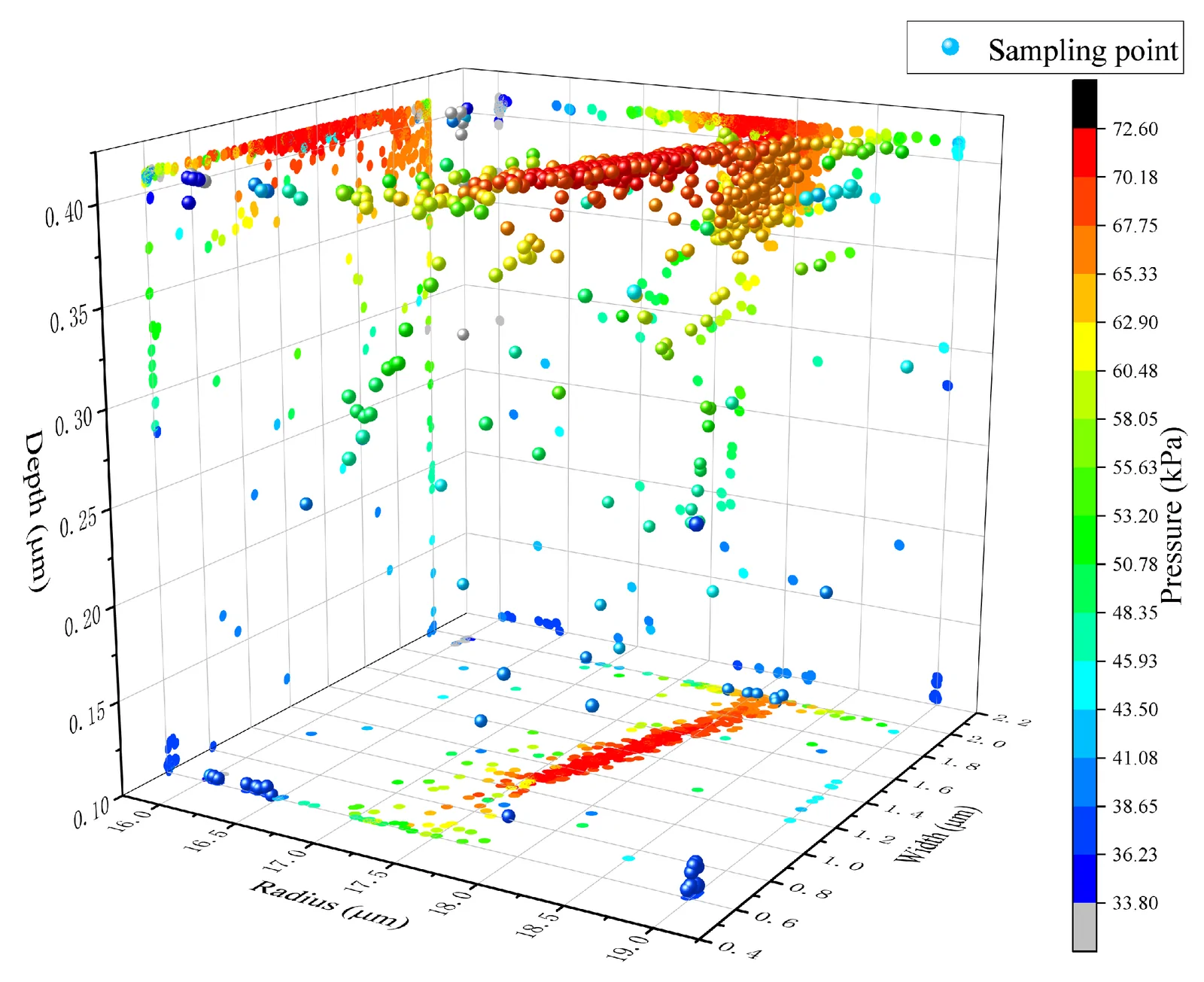
Spatial distribution of sampling points in the trench three-parameter global optimization.

**Figure 25:**
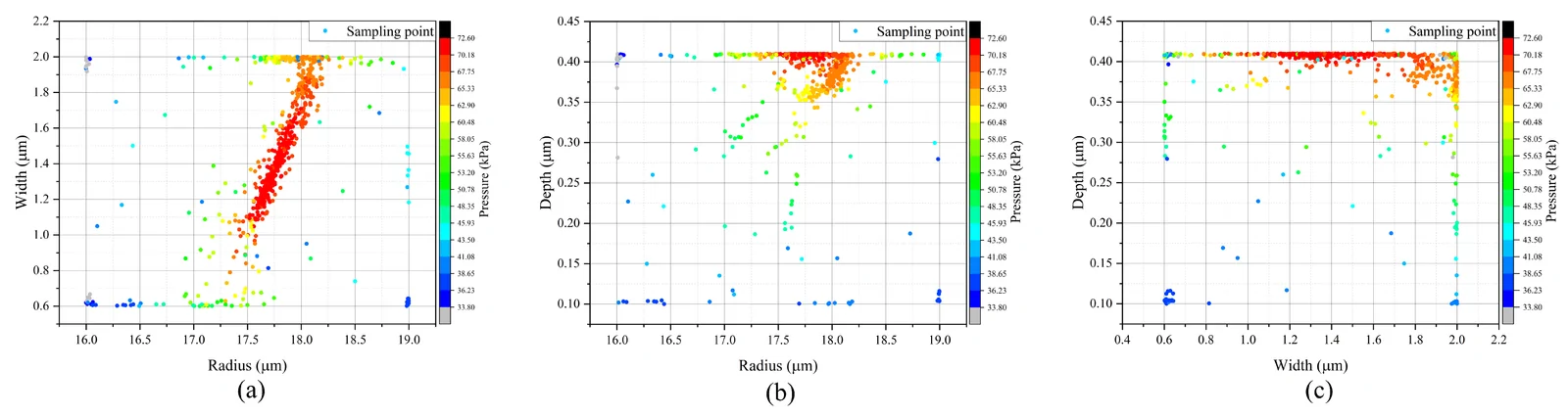
Projected distributions of sampling points onto two-dimensional planes: (**a**) radial position–width plane; (**b**) radial position–depth plane; (**c**) width–depth plane.

The distribution of 960 sampling points across the three-parameter space is depicted in Figure 24, where the color scale indicates the output acoustic pressure. Notably, the high-performance candidates (>65 kPa, indicated in red) are densely clustered within a restricted three-parameter subspace characterized by a radial position of approximately 17.3–18.5 μm, a depth of approximately 0.35–0.41 μm, and a width of approximately 0.8–2.0 μm. The sharp decline in acoustic pressure outside this cluster suggests that high performance is only achievable through a precise combination of these variables. Such a distribution confirms the existence of strong synergistic interdependencies between the parameters, validating the requirement for simultaneous three-parameter global optimization rather than individual parameter tuning. Figure 25 presents the distribution of all sampling points via projections of the three-dimensional scatter plot onto the radial position–width, radial position–depth, and width–depth orthogonal planes.

The distribution in the radial position–width plane in Figure 25a reveals that the high-performance region (>70 kPa) exhibits a distinct unimodal distribution along the radial position dimension, concentrated within a narrow range of 17.5–18.1 μm for radial position and 1.1–1.9 μm for width. When the radial position deviates below 17.3 μm or above 18.2 μm, the acoustic pressure drops sharply to below 60 kPa even if the width remains within its optimal range, indicating that radial position plays a dominant role in device performance. Its effective operating window is only approximately 0.6 μm, representing the most stringent precision requirement. Conversely, when the radial position is optimized, the acoustic pressure remains above 65 kPa for widths ranging from 1.0 to 2.0 μm. This suggests that the performance is relatively insensitive to width variations in this range, thereby offering a higher fabrication tolerance.

The sampling point distribution in the radial position–depth plane of Figure 25b indicates that the high-performance region (>70 kPa) is similarly concentrated within the radial position range of 17.5–18.1 μm and a depth range of 0.39–0.41 μm, with an increasing density as the depth approaches 0.41 μm. Within the depth interval of 0.36–0.41 μm, the acoustic pressure can be maintained above 65 kPa as long as the radial position remains optimal, suggesting a high fabrication tolerance for the depth parameter. However, when the depth is less than 0.35 μm, the acoustic pressure generally drops below 63 kPa, even with an optimal radial position. This occurs because shallow trenches have a limited impact on membrane stiffness, resulting in a constrained increase in the output acoustic pressure. The resulting band-like distribution further confirms that radial position is the primary determinant of performance.

The sampling point distribution in the depth–width plane of Figure 25c is the most dispersed among all projections. The high-performance region (>70 kPa) is scattered across a broad parameter space, specifically within a depth of 0.38–0.41 μm and a width of 0.9–2.0 μm, with the highest concentration observed in the 1.2–1.6 μm width range. However, when the depth is less than 0.35 μm, the acoustic pressure fails to exceed 63 kPa irrespective of the width, indicating a clear performance threshold where the acoustic pressure drops significantly. Consequently, the trench depth should ideally approach 0.41 μm to ensure high output acoustic pressure.

The three-parameter global optimization results indicate that radial position governs the device performance due to its high sensitivity, with an effective performance window of approximately 0.6 μm. The abrupt drop in pressure upon deviation highlights the radial position as the most critical parameter for process stability. A general positive trend exists between pressure and depth, where values approaching 0.41 μm yield superior output, aligning with the sampling point distribution shown in Figure 17. Under optimized radial and depth conditions, the width exhibits significant robustness over a wide interval, offering the greatest fabrication tolerance. This three-parameter analysis reinforces the consistency of the trends identified in the preceding single-parameter sequential studies.

**Figure 26:**
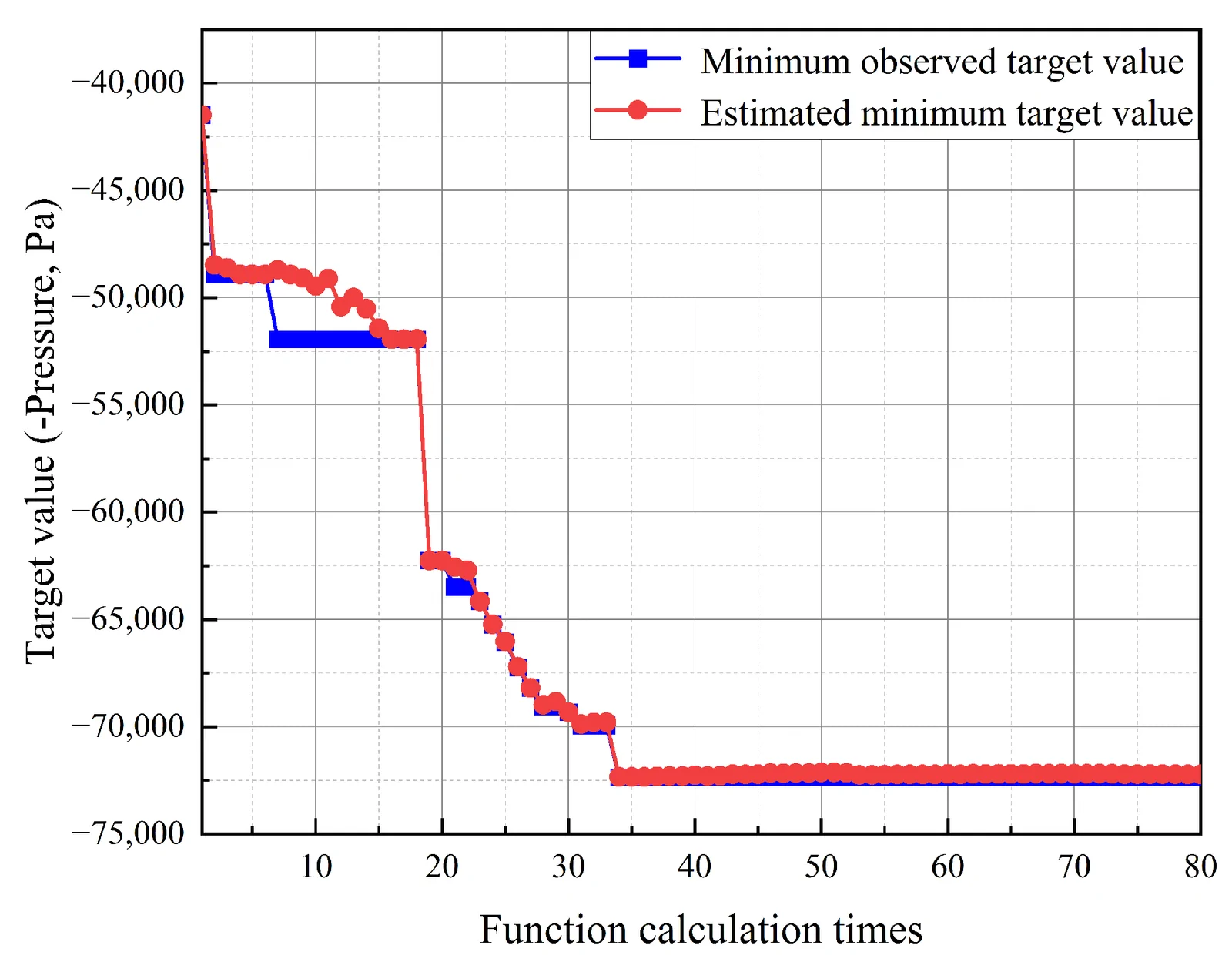
Convergence curve of the three-parameter global Bayesian optimization.

The optimal sampling point identified from 12 independent three-parameter global Bayesian optimization runs is located at a radial position of 17.70 μm, a depth of 0.41 μm, and a width of 1.31 μm, corresponding to a peak acoustic pressure of 72.34 kPa. Figure 26 illustrates the convergence process of a representative three-parameter global Bayesian optimization run. During the first 18 iterations, a Sobol sequence was employed to generate initial sampling points, ensuring uniform coverage across the three-parameter space to establish the initial training dataset for the GP surrogate model. A significant performance leap occurred at the 19th iteration, marking the successful localization of the high-performance region. Iterations 19 to 33 represent the deep optimization phase, where the observed optimal values improved steadily, the search range narrowed, and the surrogate model predictions showed high consistency with the measured values. The global optimum within the considered design space was identified at the 34th iteration, and the observed value remained constant from iterations 35 to 80. The absence of better solutions over consecutive evaluations confirms that the optimization achieved stable global convergence. This figure demonstrates that Bayesian optimization can effectively locate high-pressure regions even when three parameters vary simultaneously with inter-parameter coupling.

#### 4.3.2. Repeated Runs and Stability of Three-Parameter Global Optimization

Statistical analysis was performed on 12 optimal sampling points from repeated independent three-parameter global Bayesian optimization runs, as summarized in Table 6. The mean radial position is 17.71979 μm with a CV of 0.139%, exhibiting a fluctuation range of only approximately 0.077 μm. The CV for depth is 0.084%, the lowest among all parameters, indicating that depth possesses the highest convergence stability. Although the width shows a higher CV of 2.591%, the corresponding fluctuation in acoustic pressure is only about 105 Pa, which validates the previously discussed existence of a flat response interval near the optimal width. The mean acoustic pressure across the 12 runs is 72,289.77 Pa with a standard deviation of only 37.33 Pa (CV = 0.052%). These results demonstrate that the optimization method consistently converges to nearly identical optimal solutions regardless of stochastic initial conditions, thereby exhibiting superior engineering reliability. The clustering characteristics of high-pressure points in Figure 24 further validate the efficacy of the proposed method.

**Figure 27:**
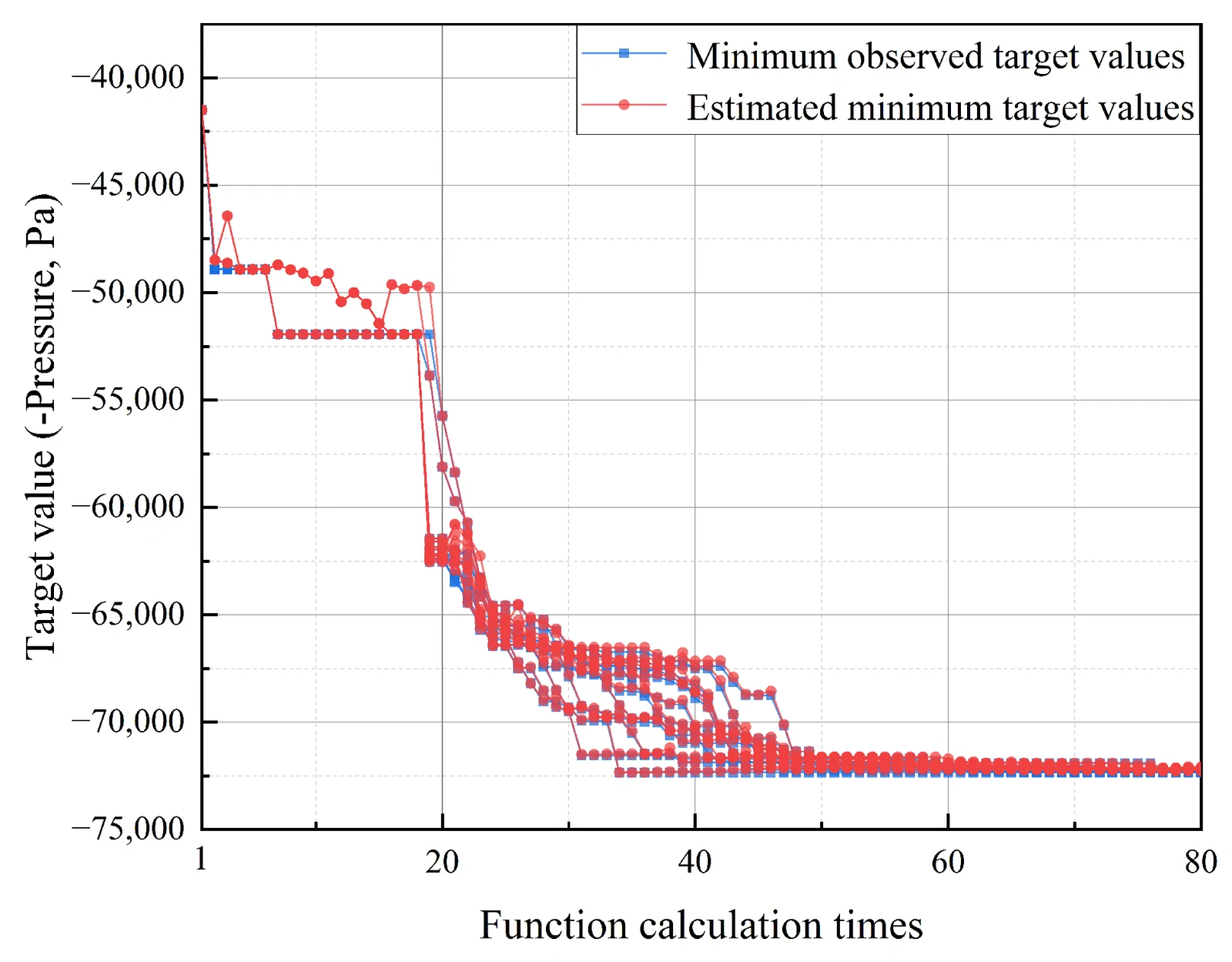
Convergence curves across 12 independent three-parameter global Bayesian optimization trials.

The convergence trajectories for 12 independent three-parameter global Bayesian optimization runs are depicted in Figure 27. By employing a Sobol sequence for the 18 initial deterministic samples, the optimization paths coincide initially before diverging during the adaptive exploration phase. The iterations required for steady-state convergence span from 34 to 77, with a mean of 64 evaluations. Following convergence, the curves plateau, signifying that no marginal performance gains are achieved. The predefined budget of 80 evaluations successfully captures the convergence inflection points for all trials, ensuring that even late-converging cases reliably identify the global optimum within the considered design space without premature termination. The pressure CV of 0.0516336% in Table 6 underscores the sufficiency of this 80-iteration limit for the three-parameter trench coupling optimization. This budget strikes an optimal balance between ensuring complete convergence and minimizing FEA computational overhead. The ability of multiple independent trials to consistently pinpoint the same global performance regime demonstrates the robust search stability and reliability of the proposed Bayesian optimization framework.

#### 4.3.3. Optimized Trenched Membrane Design

Across the 960 total sampling points generated from the 12 independent rounds of three-parameter global Bayesian optimization, the maximum output acoustic pressure was identified as 72.34 kPa. The corresponding optimal configuration was found at a radial position of 17.70 μm, a depth of 0.41 μm, and a width of 1.31 μm. This peak value, consistently approached by all independent trials, represents the global optimum within the considered design space.

**Figure 28:**
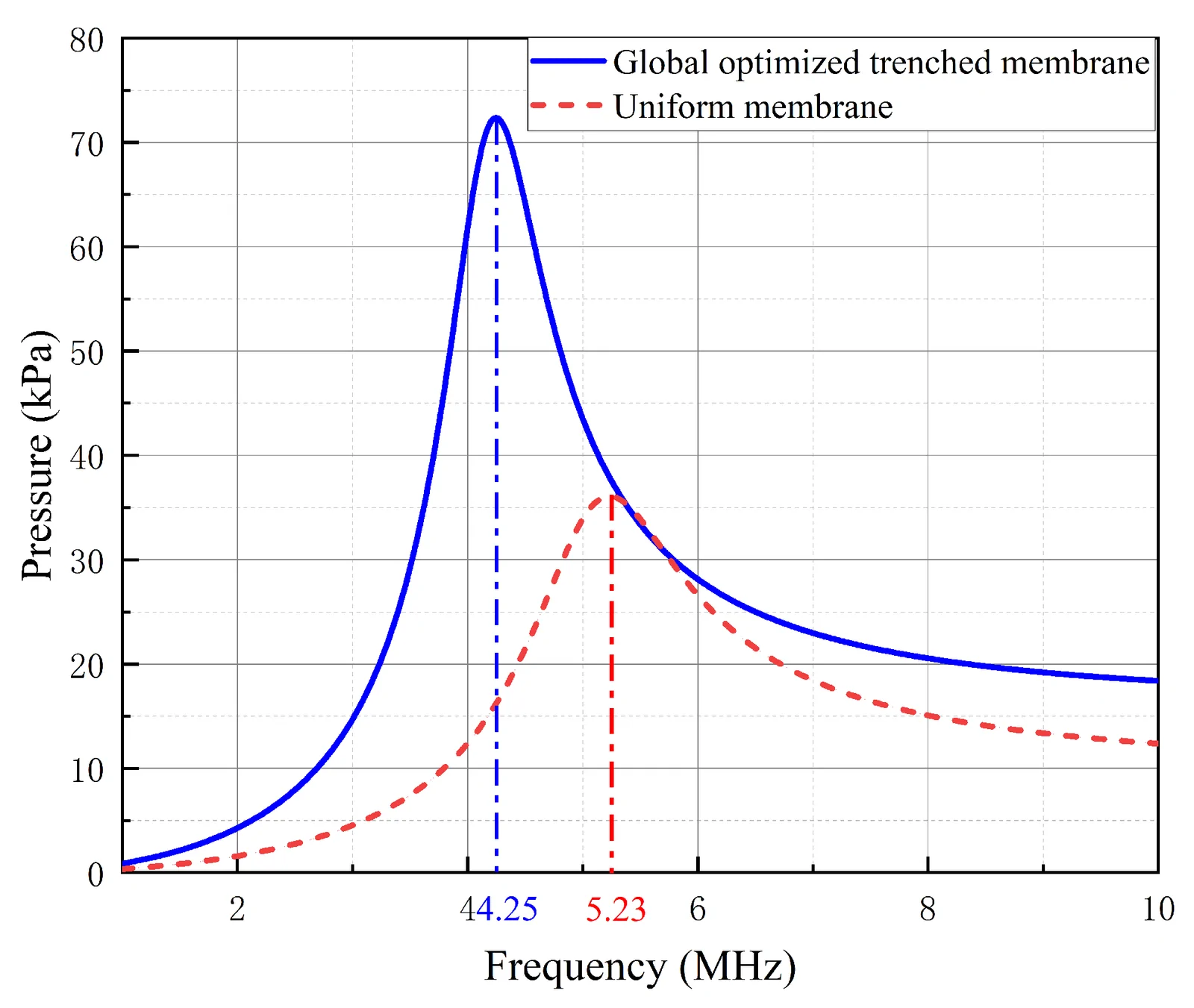
Acoustic output acoustic pressure comparison of the optimized trenched membrane CMUT and uniform membrane CMUT. The blue dashed-dotted indicates the peak frequency of 4.25 MHz for the trenched membrane CMUT, while the red dashed-dotted indicates 5.23 MHz for the uniform membrane CMUT.

Figure 28 shows the comparison of output acoustic pressure between the optimized trenched membrane CMUT and the uniform membrane CMUT under the same conditions. The trenched CMUT achieved a peak acoustic pressure of 72.34 kPa at 4.25 MHz, with a 3-dB bandwidth of 0.92 MHz. In comparison, the uniform membrane CMUT obtained a peak acoustic pressure of 36.14 kPa at 5.23 MHz, with a 3-dB bandwidth of 1.43 MHz. The output acoustic pressure of the optimized trenched CMUT was improved by 100.15%. Here, the pressure-bandwidth product is introduced as an indicator to evaluate the output acoustic pressure and bandwidth performance of the CMUTs [36]. The pressure-bandwidth products of the trenched membrane CMUT and the uniform membrane CMUT were 66.56 kPa MHz and 51.69 kPa MHz, respectively, resulting in an improvement of 28.77%. It can also be observed that, compared to the uniform membrane CMUT, the central frequency decreases when adding the trench structure to the vibrating membrane. The primary reason is that the trench structure reduces the equivalent stiffness of the membrane.

**Figure 29:**
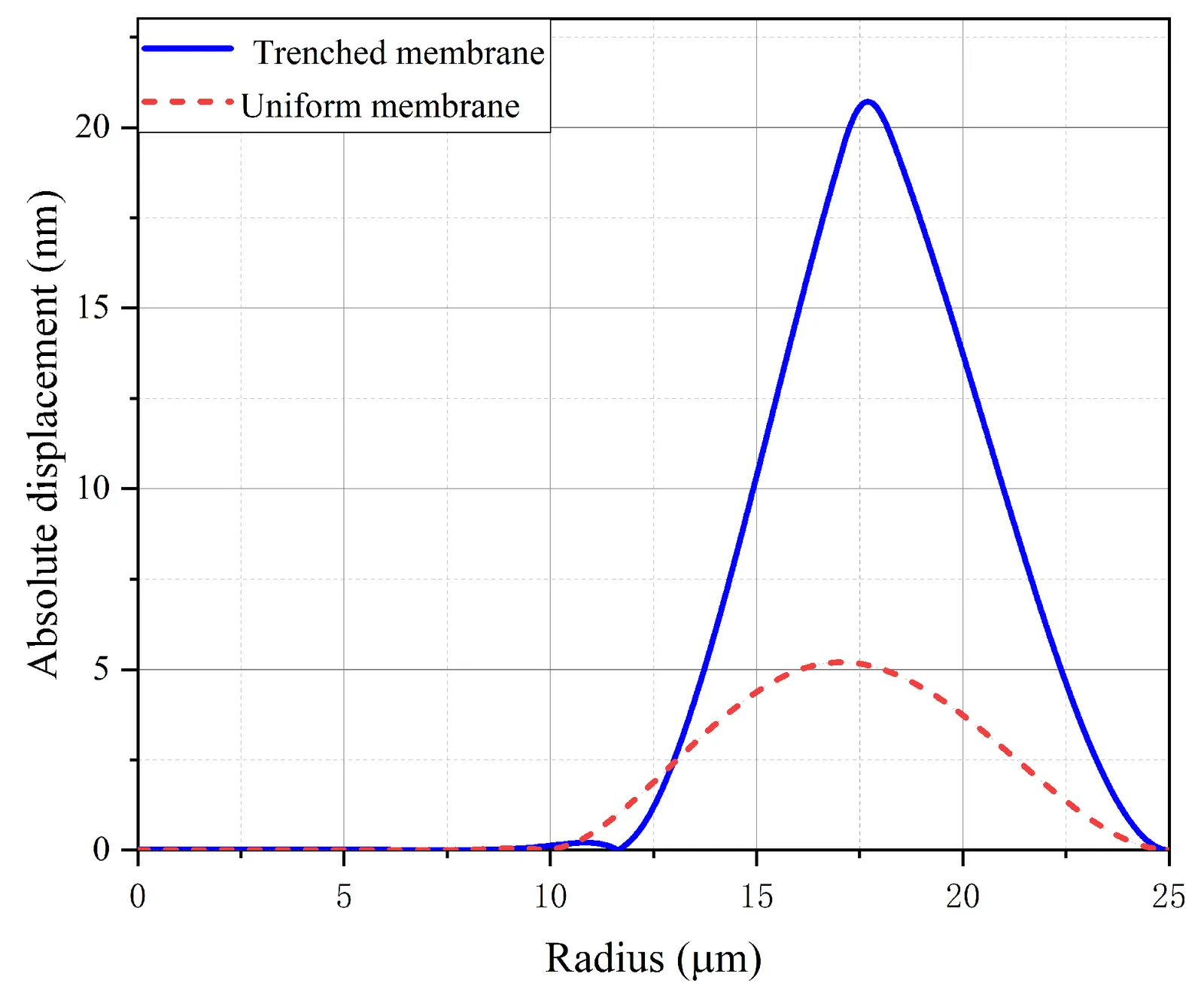
Deflection profiles of the trenched membrane and uniform membrane CMUT.

Figure 29 shows the radial distribution of membrane deflection for the trenched and uniform membrane CMUT under 130 V DC bias and excitation of 1 V AC voltage. The trenched membrane exhibits a significantly larger peak deflection (20.71 nm) at the center, which is approximately 3.98 times that of the uniform membrane (5.20 nm). Moreover, the deflection profile of the trenched membrane is more concentrated near the center, resembling a piston-like vibration, while the uniform membrane shows a broader, more diffuse deflection distribution. This improvement occurs because the trench structure makes the membrane less stiff in the vibrating area. This reduction in stiffness helps to loosen mechanical limits and allows for a more uniform and effective vibration.

To quantitatively evaluate the effective vibration output, the volume displacement (proportional to volume velocity at a given frequency) is calculated as the area-weighted integral of membrane displacement:

(35) $$V=\int_{0}^{R}w\left(r\right)\;\cdot \;2\pi r\;dr$$

The volume displacement was 14.35 $\mu m^{3}$ for the trenched membrane CMUT and 4.51 $\mu m^{3}$ for the uniform membrane CMUT, yielding a ratio of 3.18. The resonant frequency of the trenched membrane was 4.25 MHz, which was lower than the 5.23 MHz of the uniform membrane. Nevertheless, the substantial increase in volume displacement fully compensates for the frequency reduction, resulting in a larger volume velocity and thus a higher output acoustic pressure.

## 5. Discussion

### 5.1. Advantages of Bayesian Optimization in CMUTs Design

Theoretical and FEA simulations indicate that introducing a trench on the membrane of a collapse-mode CMUT can significantly enhance the output acoustic pressure. The trench structure involves three spatial parameters of radial position, depth, and width. However, there is no explicit analytical relationship exists between these three parameters and the resulting acoustic pressure. If traditional grid search methods were used, optimizing the combinations of the three parameters could require up to thousands of FEA simulations, resulting in significant consumption of computational resources and a high likelihood of getting trapped in local optima.

Bayesian optimization is a global optimization method designed for black-box functions, particularly suited to evaluating costly objective functions that lack analytical expressions. Its core advantage lies in its ability to approximate the global optimum with very few evaluations, typically in the range of tens to a few hundred [37]. This method constructs a probabilistic surrogate model of the objective function and intelligently balances exploration and exploitation through an acquisition function. It significantly outperforms traditional grid search or random search in terms of sample efficiency, uncertainty quantification, and handling complex constraints.

In this study, to further enhance the efficiency of Bayesian optimization in trenched membrane design, the mechanical behavior and acoustic radiation characteristics of the annular vibrating membrane in the collapse mode were first analyzed using von Kármán large-deflection plate theory. This analysis identified the positions on the membrane that contribute most significantly to acoustic radiation, allowing for an estimation of the optimal radial position for the trench. Based on this theoretical estimation, the search space for the trench’s radial position was narrowed from [10 μm, 25 μm] to [16 μm, 19 μm], effectively reducing the search space to 20% of its original range. Consequently, for the same total number of iterations, the sampling density in the high-potential region is increased, leading to significantly faster convergence. For the single-parameter sequential optimizations, the radial position, depth, and width required only 8, 7, and 8 simulation iterations, respectively, to reach convergence, all of which are less than half of the preset total iteration number of 24. Based on these findings, a three-dimensional global optimization was further conducted to jointly optimize the trench radial position, depth, and width. The three-parameter optimization, consisting of 80 evaluations, achieved a higher output pressure than the sequential single-parameter approach (72.34 kPa vs. 70.02 kPa), confirming that the three parameters are strongly coupled and their synergistic interactions cannot be captured by sequential single-parameter approaches. This “theory-guided Bayesian optimization” strategy provides a simple and effective starting point for trench design in collapse-mode CMUTs and can be extended to the optimization design of other CMUT structural parameters.

### 5.2. Comparison of Different CMUT Structures for Pressure Enhancement

This section compares the optimized collapse-mode trenched CMUT with other structural modification strategies reported in the literature for enhancing output pressure, such as embossed membranes, annular electrodes, stepped cavities, and center-mass structures. A comprehensive comparison of these structures is presented in Table 7.

The trenched CMUT exhibits a high acoustic pressure output due to its operation in a collapsed mode. Both the trenched membrane and embossed membrane CMUTs operate in collapsed mode. Apart from the specific structural differences between the trench and embossed patterns, all other parameters in the FE models, including dimensions, materials, DC bias, and excitation AC signals, are identical to facilitate a fair comparison. The results demonstrate that the trenched membrane CMUT achieves a 6.67% increase in output acoustic pressure compared to the embossed membrane structure.

### 5.3. Frequency Shift and Bandwidth Variation

As shown in the previous section, the design of the trench structure results in a significant increase in output acoustic pressure. However, this enhancement is accompanied by a decrease in center frequency, which shifts from 5.23 MHz to 4.25 MHz, along with a narrowing of the bandwidth. The downward shift in center frequency is primarily due to the localized reduction in bending stiffness induced by the trench. According to plate theory, the flexural rigidity *D* is proportional to the cube of the local thickness, and the trench design effectively reduces the overall mechanical stiffness of the vibrating membrane. Although the trench simultaneously removes some mass from the structure, the stiffness reduction dominates the dynamic response during collapse-mode operation, resulting in a decrease in the center frequency.

In conventional abdominal ultrasound diagnostics, frequencies of 5 MHz and below are typically sufficient to meet most clinical requirements [38]. For organs such as the liver, pancreas, and kidneys, standard convex array probes generally operate within the 2–5 MHz range. This frequency band provides good penetration and acceptable resolution for typical adults. The optimized frequency of 4.25 MHz remains well within this range, ensuring full compatibility with existing imaging systems. More importantly, the increase in acoustic pressure, which directly enhances the signal-to-noise ratio (SNR) and penetration depth, is achieved at a clinically relevant frequency. While the center frequency and fractional bandwidth of the trenched membrane CMUT slightly reduce, leading to a minor decrease in axial and lateral resolution, the output acoustic pressure increases by over 100%, resulting in a signal gain of +6 dB. Since the acoustic attenuation coefficient of human tissue is proportional to frequency, and considering that abdominal imaging involves deep tissue assessment, the intensity of echo signals tends to diminish due to attenuation. Consequently, the combination of doubled output acoustic pressure and the resulting reduction in attenuation at the lower frequency typically leads to an enhancement in overall image SNR. This ultimately means that details in deeper regions of the image become clearer, thereby improving far-field imaging performance.

In the design of trenched membrane CMUT devices, it is essential to select appropriate dimensions and operating points based on the specific application of the ultrasound probe, while also optimizing the trench structure. Furthermore, for devices operating in collapse mode, the center frequency is primarily determined by the width of the vibrating membrane. Consequently, the center frequency of the collapse-mode CMUT can be tuned by adjusting the DC bias voltage. Although an optimal trench position exists, Figure 16 demonstrates that for a fixed trench configuration, the output acoustic pressure remains enhanced relative to the uniform membrane CMUT across a DC bias range of 110 V to 150 V. Specifically, within the range of 120 V to 140 V, the pressure enhancement exceeds 57%.

### 5.4. Proposed Fabrication Process

#### 5.4.1. Fabrication Process

**Figure 30:**
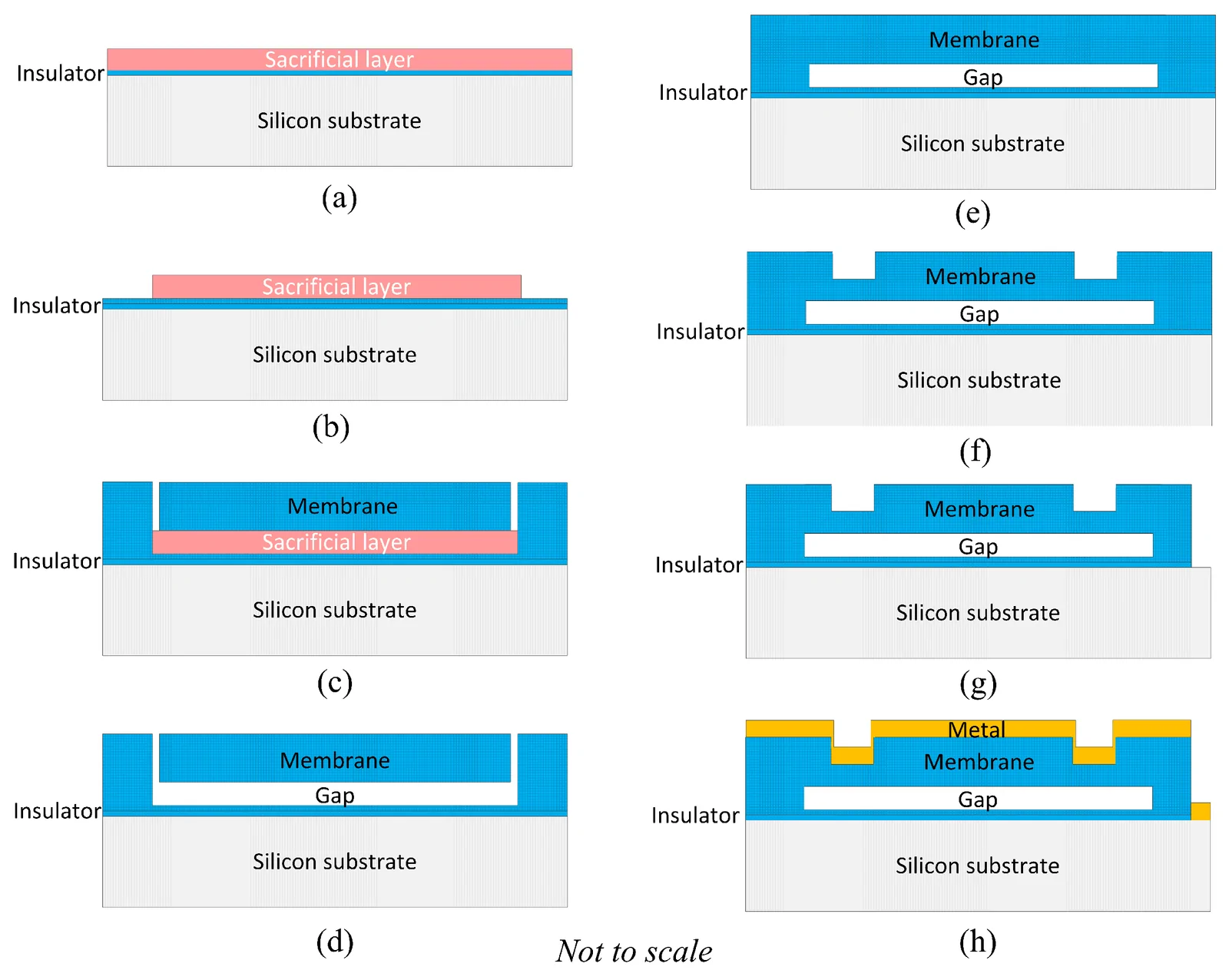
Proposed fabrication process for trenched membrane CMUTs. (**a**) Sacrificial layer deposition; (**b**) forming cell pattern on sacrificial layer; (**c**) etching pathways to the sacrificial layer; (**d**) creating the gap; (**e**) sealed vacuum gap; (**f**) forming trench on membrane; (**g**) bottom electrode area exposed; (**h**) pattern electrodes and connections.

A feasible fabrication process for the trenched membrane CMUT is presented in Figure 30. Similar to our previously developed embossed pattern CMUT fabrication process [39], this is a customized six-mask sacrificial release technique. First, on an N-type heavily doped silicon wafer, SiO_2_ is grown using dry oxidation, followed by the deposition of Si_3_N_4_ as an insulating layer using the low-pressure chemical vapor deposition (LPCVD) method, with polysilicon serving as the sacrificial layer, as shown in Figure 30a. Polysilicon is then etched with Mask #1 and reactive ion etching (RIE) to create the shape of the circular CMUT cell cavity and the etching channels, as illustrated in Figure 30b.

Next, low-stress Si_3_N_4_ is deposited using PECVD or LPCVD as the structural layer and membrane. With Mask #2 and RIE, etching holes are formed in the release channels surrounding the CMUT cell, creating pathways leading to the polysilicon, as depicted in Figure 30c. The wafer is then immersed in potassium hydroxide (KOH) solution, and wet etching is performed to completely release the polysilicon sacrificial layer from the release holes, forming the cavity structure, as shown in Figure 30d.

Following this, PECVD is used to fill the etching holes by depositing Si_3_N_4_. Subsequently, Mask #3 and RIE are employed to thin the membrane, restoring its thickness to the initial value, which ultimately seals the cavity, as illustrated in Figure 30e. Using Mask #4 and RIE, an annular trench is etched onto each CMUT’s circular membrane, forming the trench structure shown in Figure 30f.

After that, Mask #5 and RIE are used to completely etch away the Si_3_N_4_ and SiO_2_ in the bottom electrode pad region, creating pathways to the heavily doped silicon substrate, as shown in Figure 30g. Finally, a metal layer is deposited using e-beam evaporation, and Mask #6 is applied with dry or wet etching method to form the top electrode pad, bottom electrode pad, and connections, as shown in Figure 30h.

#### 5.4.2. Fabrication Considerations

According to the global three-parameter optimization analysis, the key to enhancing the output pressure of trenched collapsed-mode CMUTs lies in the synergistic optimization of the trench’s radial position, depth, and width, rather than the independent contribution of a single geometric parameter. Consequently, the impact of fabrication tolerances and material properties on the performance enhancement must be evaluated during the device manufacturing process. The enhancement of acoustic pressure is highly sensitive to the radial position of the trench, which features a narrow optimal window. This imposes a stringent requirement on alignment precision during fabrication. In contrast, the trench width exhibits high process redundancy, as high output pressure can be maintained across a relatively broad range. Furthermore, the magnitude of pressure enhancement is positively correlated with the trench depth. However, it indicates that when the trench depth exceeds 0.43 μm in a 0.60 μm thick membrane—corresponding to 72% of the thickness and a residual thickness of less than 0.17 μm—the membrane vibration enters a nonlinear regime. It results in an abrupt jump followed by a decay in output pressure, which may compromise device stability. Therefore, the etching depth must be strictly controlled during fabrication to avoid approaching this critical threshold.

Membranes are fabricated using either PECVD or LPCVD processes, where the membrane thickness directly governs the center frequency, output acoustic pressure, and collapse voltage. To mitigate the impact of deposition non-uniformity, it is recommended to select dies from the central region of the wafer, where thickness uniformity is better. Dielectric charging represents a critical reliability bottleneck for collapsed-mode CMUTs during long-term operation. Under identical conditions, LPCVD silicon nitride exhibits significantly superior resistance to dielectric charging compared to PECVD nitride due to its lower hydrogen content and charge trap density, making it more suitable for continuous operation. While residual stress in both types of silicon nitride can be maintained within reasonable limits by tuning deposition parameters, geometric discontinuities at the trench roots and sharp corners induce significant stress concentration. These localized stresses may far exceed bulk levels, potentially compromising mechanical integrity over the long term. In contrast, LPCVD silicon nitride offers a distinct advantage in mechanical reliability for trenched structures, owing to its superior compactness and fracture toughness.

In summary, the device design and process implementation of trenched collapsed-mode CMUTs require a strategic balance between performance enhancement and process feasibility, taking into full account the combined effects of etching tolerance, thickness uniformity, dielectric charging, and trench-induced stress concentration.

### 5.5. Limitations and Outlook

Although this study has achieved an optimal trenched membrane collapse-mode CMUT structure with high output acoustic pressure using Bayesian optimization and FEA simulation, there are still certain limitations. First, the theoretical model relies on simplified assumptions. The von Kármán large-deflection plate theory guides the prior search space for Bayesian optimization, which accounts for geometric nonlinearity under large static deflection. However, when the CMUT operates under high AC excitation or in a deep-collapse mode, the deformation of the membrane may further exceed the applicability of the current theoretical model. In such cases, discrepancies may arise between the predicted maximum deflection position and the actual value.

Second, the surrogate model in Bayesian optimization depends on the accuracy of the FEA simulation. In this study, the fitting of the GP is based entirely on data derived from FEA simulations, which utilize idealized material parameters, ideal clamped boundary conditions, and linear small-deformation assumptions. In reality, CMUT devices often incorporate non-ideal factors during fabrication, such as residual stress gradients and variations in membrane thickness, which are challenging to model accurately in simulations. Consequently, there may be a discrepancy between the optimal trench parameters obtained from simulations and actual devices.

Third, the proposed trenched CMUT design has not yet been experimentally validated. While the FEA model employed in this study is developed from the same framework as our previously validated embossed CMUT model [22,23], the specific trenched structure requires direct experimental verification. The absence of fabricated device measurements means that the predicted performance enhancement remains to be confirmed through physical testing.

Future research will focus on fabricating trenched membrane CMUT devices based on the process proposed in this study. Experimental tests will be conducted to evaluate their vibration characteristics, center frequencies, and acoustic pressure outputs, thereby validating the effectiveness of the FEA model. In addition, further multi-objective optimization studies will be conducted to comprehensively balance key factors such as acoustic performance, structural reliability, and process tolerances.

## 6. Conclusions

This study presents a systematic approach to enhance the output acoustic pressure of collapse-mode CMUTs by optimizing trench geometry. An analytical model based on von Kármán large-deflection plate theory was established to estimate the optimal trench radial position, which was then used to define the search space for Bayesian optimization. A three-dimensional global Bayesian optimization framework was subsequently employed to globally optimize the trench radial position, depth, and width. The analytical prediction of the optimal trench position is validated by the results of both single-parameter sequential and three-parameter global Bayesian optimizations, which yielded a high degree of consistency with a deviation of less than 1.15% (17.50 μm vs. 17.70 μm). This confirms that the theoretical model effectively captures the radial trend of the volume-velocity integrand and provides a reliable starting point for optimization.

The three-parameter joint optimization reveals strong synergistic interactions among the radial position, depth, and width, achieving a higher output pressure than sequential single-parameter approaches (72.34 kPa vs. 70.02 kPa). The consistency between the analytical prediction and the numerical optimization, together with the superior performance of the three-dimensional joint optimization, demonstrates that integrating physical modeling with data-driven optimization offers an efficient and reliable pathway for CMUT structural design. The optimized trenched membrane CMUT demonstrated a 100.15% increase in output acoustic pressure compared to the uniform membrane CMUT, along with a 28.77% enhancement in the pressure-bandwidth product.

Future work will focus on the fabrication and experimental characterization of the optimized trenched CMUT, including the evaluation of its vibration characteristics, center frequency, and output acoustic pressure. Further multi-objective optimization studies will also be conducted to comprehensively balance acoustic performance, structural reliability, and process tolerances.

## Figures and Tables

**Table 1:** The optimum positions from analytical and FEA methods.

$\eta_{c}$	Analytical (μm)	FEA (μm)	Error (%)
0.3	16.25	16.75	−2.65
0.4	17.50	17.75	−1.41
0.5	18.75	19.00	−1.32
0.6	20.00	20.22	−1.09

**Table 2:** Dimensions of the CMUT cell in FEA simulation.

Model Dimension	Unit (μm)
Membrane radius	25
Membrane thickness	0.6
Gap height	0.4
Insulator thickness	0.11
Water medium radius	150

**Table 3:** Material parameters in simulation.

Material Parameters	Value
Young’s modulus of Si_3_N_4_	110 GPa
Poisson’s ratio of Si_3_N_4_	0.27
Dielectric permittivity of Si_3_N_4_	5.4
Density of Si_3_N_4_	3100 kg/m^3^
Dielectric permittivity of SiO_2_	3.7

**Table 4:** Hyperparameters of the Bayesian optimization algorithm.

Hyperparameter	Definition	Value
IsObjectiveDeterministic	Deteministic objective function	False (default)
ExplorationRatio	Propensity to explore	0.5 (default)
GPActiveSetSize	Fit Gaussian process model to GPActiveSetSize or fewer points	300 (default)
UseParallel	Compute in parallel	False (default)
MaxObjectiveEvaluations	Objective function evaluation limit	24 (single-parameter) /80 (three-parameter)
MaxTime	Time limit	100 × 3600 s
NumSeedPoints	Number of initial evaluation points (random for single-parameter; Sobol sequence for three-parameter)	4 (single-parameter)/ 18 (three-parameter)
Kernel Function	Gaussian process kernel	ARD Matérn 5/2 kernel

**Table 5:** Statistics of the trench’s radial position, depth, and width across 12 single-parameter sequential Bayesian optimization trials.

Parameter	Item	Mean	SD	CV	Minimum	Median	Maximum
Position	Position (μm)	17.47613	0.00947	0.05417%	17.46092	17.47507	17.49385
	Pressure (Pa)	68,369.36533	96.91633	0.142%	68,282.67054	68,337.97643	68,518.43957
Depth	Depth (μm)	0.44074	0.00336	0.762%	0.437	0.44018	0.44743
	Pressure (Pa)	72,859.56481	979.56442	1.344%	71,957.88892	72,469.34739	74,809.86781
Width	Width (μm)	0.98807	0.01634	1.654%	0.95248	0.98533	1.01234
	Pressure (Pa)	69,939.58821	54.3996	0.07778%	69,828.61396	69,937.02876	70,020.4081

**Table 6:** Statistics of trench’s radial position, depth and width across 12 three-parameter global Bayesian optimization trials.

Parameter	Mean	SD	CV	Minimum	Median	Maximum
Position (μm)	17.71979	0.02457	0.139%	17.68396	17.7202	17.76048
Depth (μm)	0.40949	0.000342729	0.0836956%	0.40892	0.4096	0.40991
Width (μm)	1.32354	0.03429	2.591%	1.25151	1.32226	1.36369
Pressure (Pa)	72,289.77166	37.32584	0.0516336%	72,238.7042	72,304.54237	72,343.91761

**Table 7:** Comparison of output pressure and pressure enhancement across different CMUT structures.

Structure	Pressure	Improvement	Operation Mode	Remark
This work	72.34 kPa/V	100.15%	Collapse	FEA
Embossed membrane [22]	67.9 kPa/V	88.1%	Collapse	FEA
Annular electrode [16]	∼130kPa	270%	Conventional	FEA, in air
T-shaped cavity [17]	6.96 kPa/V	56.1%	Conventional	FEA, in air
Dual-Layer CMUT [21]	∼31kPa/V	17.7%	Conventional	FEA
Annular electrode and membrane grooves [19]	227 kPa	39.3%	Conventional	Device experiment
Indirectly clamped [20]	191 kPa	72.1%	Conventional	Device experiment
Center Mass [10]	5.75 kPa/V	82.5%	Conventional	Device experiment

## Data Availability

Dataset available on request from the authors.

## Abbreviations

The following abbreviations are used in this manuscript:

MEMSs	micro-electro-mechanical systems
CMUTs	capacitive micromachined ultrasonic transducers
CV	coefficient of variation
PZT	lead zirconate titanate
POCUS	point-of-care ultrasound
PMUTs	piezoelectric micromachined ultrasonic transducers
FEA	finite-element analysis
FE	finite element
PECVD	plasma-enhanced chemical vapor deposition
GP	Gaussian process
SDs	standard deviations
SNR	signal-to-noise
LPCVD	low-pressure chemical vapor deposition
RIE	reactive ion etching
KOH	potassium hydroxide
